# Real-Time Working-Point Detection and Estimation for an Evisceration Robot Using a Single Time-of-Flight Camera and a Shared-Backbone Multi-Task Network

**DOI:** 10.3390/s26216884

**Published:** 2026-10-30

**Authors:** Donggoo Lee, Hyunmin Oh, Minwoo Ryu, Kyounghoon Lee, Jeonghun Kim

**Affiliations:** R&D Center, ROBOS Co., Ltd., Seongnam 13487, Republic of Korea; billy.lee@xrobos.ai (D.L.); felix.ohm@xrobos.ai (H.O.); marcus.yoo@xrobos.ai (M.R.); kyounghoon.lee@xrobos.ai (K.L.)

**Keywords:** DINOv2, evisceration, hand–eye calibration, keypoint estimation, multi-modal sensing, multi-task network, real-time perception, slaughter automation, time-of-flight camera, working-point estimation

## Abstract

Evisceration is a slaughter stage with strong demand for automation. The robot must detect four working-point classes and estimate each working point in pixels. We compose the network input from the luminance and depth images of a single Time-of-Flight (ToF) camera, avoiding the registration error of RGB-D cameras, and propose a retroreflective spherical-marker target for hand–eye calibration; the calibration procedure built on it was 3.1 and 3.3 times more repeatable in translation and rotation than a grid-target procedure. We extend the RF-DETR-L detector into a 43.9 M-parameter multi-task network in which a keypoint branch initialized from the detection outputs shares the DINOv2-S backbone. On five carcasses from the same facility not used for training (500 images, three runs), the network reached a detection AP of 68.6 and a keypoint AP of 73.3, comparable to the serial detector–pose-estimator pipeline it replaces (68.3 and 73.2), with a mean working-point error of 1.83 vs. 2.01 px. With TensorRT FP16, its network-stage latency was 2.34 ms (plus 3.80 ms resize), compared with 4.11 ms for the serial pipeline (3.40 ms batched), and stayed nearly constant when up to sixteen objects were forced (serial: 17.22 ms); a single-stage YOLOv8l-pose gave similar AP and network-stage latency.

## 1. Introduction

### 1.1. Research Background

Evisceration is the stage of separating and extracting internal organs from an opened carcass during slaughter. It is a high-risk operation involving cutting knives and requires high proficiency because the posture, size, and organ arrangement vary for each carcass. Furthermore, damaged organs cause the intestinal contents to contaminate the carcass, directly affecting the hygiene rating and food safety, thereby requiring both precision and consistency of the operation [1]. Thus, evisceration is one of the processes with the highest demand for robotic replacement in the automation of pig, cattle, and poultry slaughterhouses. However, owing to variations in the relative positions of the individual carcass and its internal organs, it is difficult to assign fixed cutting parameters and handle internal organs without causing damage, making evisceration a representative process that has yet to be fully automated [2]. Therefore, visual perception is required before robot control to reliably identify the working reference positions, which vary among individual carcasses.

Studies on poultry evisceration robots have reported that recognizing the organ region in RGB images of carcasses on a conveyor guides the grasping position of the manipulator, with recognition accuracy directly determining the level of organ damage and retention [3]. However, these early studies were based on classical image processing or single-task segmentation, making it difficult to simultaneously achieve robustness against the large shape variations in carcasses and real-time performance that satisfies the line takt time. Accordingly, highly accurate and high-speed deep learning-based perception models have recently gained attention in the meat production industry, with studies targeting both accuracy and speed, such as applying YOLO-family detectors to detect carcass contamination [4] or accelerating poultry part segmentation with a lightweight network (1.89-fold) [5].

### 1.2. Limitations of the Sensor Configuration

Three-dimensional perception in robotic systems is commonly performed by recognizing objects in RGB images using an RGB-D (RGB+Depth) camera, which reads the corresponding 3D points from the depth image and transmits them to the robot. This essentially requires registration between the physically separated RGB camera and depth camera. However, registration errors are inevitable due to parallax between sensors, calibration errors, and other factors. Commercial RGB-D cameras are shipped with a lower accuracy compared to that required for robotics [6]; furthermore, the factory-set alignment is also subject to change during use due to optical image stabilization and other factors [7]. During transmission of the 3D coordinates of a specific point in the RGB image to the robot, the registration error directly propagates to a control error, making it difficult to utilize for real-time control.

This paper avoids this problem by design by using only a single Time-of-Flight (ToF) camera. ToF cameras can simultaneously acquire depth and luminance images. A ToF camera emits modulated infrared light and computes the distance at each pixel from the phase delay of the reflected light; the amplitude of the same reflected signal forms the luminance image [8]. Because both images are acquired from the same pixels using the same optical system, they are free from registration errors such as those between RGB and depth images, and recognition directly on ToF luminance and depth images has been demonstrated in other domains [9,10,11,12] (Section 2.1).

The resolution of the ToF sensor used here (640 × 480) is low compared with that of color cameras, which, combined with the sensor’s inherent characteristics, makes feature localization on conventional calibration targets less reliable (Section 3.3). In vision-based robotic automation systems, calibration precision directly affects the final task accuracy. Therefore, this work also proposes a hand–eye calibration method applicable to a standalone ToF system.

### 1.3. Limitations of the Perception Network

The conventional top-down (two-stage) approach separates detection and keypoint estimation into different networks: the bounding-box region extracted by the detector is cropped and normalized to a fixed size, a pose estimator then estimates the keypoints within the cropped region, and the resulting coordinates are transformed back to the original image coordinate system (Figure 1a). This structure has three well-known drawbacks [13,14,15]; which of them matters depends on the object size and the takt time of the application, and we state below which one this work targets.

First, unidirectional error propagation. Because keypoint estimation depends entirely on the detector output, bounding-box position and size errors, as well as missed detections, are directly propagated as keypoint errors without correction.

Second, precision loss due to crop-and-resize. The top-down approach resamples the cropped region to a fixed resolution, while heatmap-based estimators typically estimate keypoints at a lower resolution than the input. When the object is larger than the crop resolution, the transformation back to the original image magnifies the estimation error by the scale factor of the crop, which can make pixel-level precision difficult to secure; when the object is smaller than the crop resolution, as for the working-point objects of this task, whose bounding boxes span a few tens of pixels in the 640 × 480 image and are resampled to the fixed input resolution of the pose estimator in the serial pipeline compared here, the crop is upsampled and no such loss arises, which is consistent with the comparable working-point error observed in Section 4.2.

Third, duplicated cost in training and operation. The two networks are trained, deployed, and executed separately, so the backbone computation is incurred twice per frame and the keypoint latency grows with the number of detected objects. As noted above, the second drawback does not arise for the working-point objects of this task, and the first is not examined here; the drawback this work targets and quantifies is the third (Section 4.3). The accuracy of the joint network relative to the serial pipeline is reported in Section 4.2.

To address similar limitations, YOLOv8 provides a multi-task configuration that adds segmentation and pose-estimation branches to the detection head [16]. A multi-task segmentation–classification network that shares a lightweight backbone has also been proposed for image analysis in edge environments [17].

### 1.4. Overview of the Proposed System

This paper proposes a visual perception system that simultaneously addresses sensor registration errors and the structural limitations of top-down networks. The system uses only a single-ToF camera as input, which eliminates by construction the registration errors between heterogeneous sensors that arise in RGB-D configurations because depth and luminance are read from the same pixels, and it integrates detection and keypoint estimation into a single multi-task network with a shared backbone. Figure 1 compares the conventional configuration, in which the detection and keypoint models are connected in series (a), with the proposed single-network configuration (b).

As shown in Figure 1b, the Proposed Network uses a single DINOv2 [18]. Small backbone, pre-trained via self-supervised learning, with three heads serving different purposes (the third, an optional instance segmentation head, is disabled in this work). The detection path outputs bounding boxes and classes through a deformable-attention-based [19] detection transformer (DETR) decoder [20], preceded by a Multi-Scale Projector (MSP) that fuses features from four backbone depths (blocks 3/6/9/12) in an all-to-all manner. Based on the keypoint–segmentation feature pyramid network (KPT·Seg FPN; cf. [21]), which constructs a multi-resolution pyramid using the same four depth features, the keypoint path estimates the class-specific working points using a deformable decoder that utilizes the center coordinates of the detection results as initial query values. Because the keypoint queries are initialized from the detection outputs (box centers), the outputs of both tasks are obtained in a single inference pass, unlike the serial configuration shown in Figure 1a.

### 1.5. Major Contributions

In summary, this paper shows that a single-ToF camera can provide both the hand–eye calibration and the image-space working-point estimation of an evisceration robot: its luminance image calibrates the camera to the robot with 0.374 mm translational and 0.022° rotational repeatability, and its luminance–depth composite drives one 43.9 M-parameter network that returns the four working-point classes. On five carcasses not used for training, the working-point error of this network was comparable to that of the two-model pipeline it replaces (1.83 vs. 2.01 px), and after TensorRT 16-bit floating-point (FP16) conversion, its network stage took 2.34 ms, nearly independent of the number of objects, compared with 4.11 ms for the two-model pipeline on the deployed path. The specific contributions are as follows.

#### 1.5.1. Single-ToF Input Configuration

We present a method of constructing a three-channel image by normalizing the depth within the working distance range and luminance within two different ranges into 8-bit values. By excluding color cameras, this approach eliminates, by construction, the inter-image registration errors of RGB-D configurations, while allowing detectors designed under the assumption of three RGB channels to be applied without modification.

#### 1.5.2. Hand–Eye Calibration in a Low-Resolution Single-ToF Environment

We propose a retroreflective spherical marker as the calibration target because it can be stably detected in low-resolution luminance images. In repeated experiments in which only the target, its detector, and the exposure setting were changed while the robot and camera remained unchanged, the calibration procedure built on the proposed marker achieved translational and rotational repeatabilities of the camera pose 3.1 and 3.3 times better, respectively, than those of the procedure built on the Radon board (0.374 vs. 1.157 mm and 0.022° vs. 0.073°).

#### 1.5.3. Integrated Network with a Shared Backbone

We propose an integrated detection and keypoint network (43.9 M) that shares a DINOv2-S backbone. It combines KPT·Seg FPN, which directly constructs multi-resolution features from intermediate layers of the backbone, with object-center-based keypoint query initialization, and it has fewer parameters than the Serial Network it replaces (85.7 M in total: 33.6 M for RF-DETR-L and 52.1 M for the four class-wise RTMPose-M models). The keypoint branch follows the ED-Pose scheme [13] of initializing keypoint queries from the detection results; it differs in estimating one class-specific working point per object, initialized on the stride-4 feature map (P2, Section 3.5) and refined with a 17-bin integral head (Section 3.9) over three pyramid levels. The contribution of these individual components was not isolated by ablation (limitation (vi) in Section 5).

#### 1.5.4. Model Compression and Verification for Real-Time Deployment

We present a deployment pipeline that converts the Proposed Network into a TensorRT FP16 engine and evaluates the engine against the PyTorch model on the same test frames. After conversion, the average precision (AP) changes by at most 0.2 points and the mean working-point error is unchanged, while the network-stage latency decreases from 17.87 to 2.34 ms, well below the 33.3 ms frame interval of the 30 fps camera, and stays nearly constant as the number of objects increases (all keypoint queries are decoded in one pass); the 2.34 ms covers the network stage only, and the resize and normalization to 704 × 704, excluded from it, took a further 3.80 ms (Section 4.3).

## 2. Related Work

### 2.1. Single-ToF Camera Systems

Studies have been conducted to directly utilize the infrared (IR) luminance images of ToF sensors as inputs for recognition models, rather than as auxiliary information for depth reliability evaluation. Sharma et al. used only luminance and depth from a single-ToF camera and achieved segmentation accuracy comparable to that obtained using both RGB and depth [9]. Yang et al. detected objects and classified materials by combining depth and luminance [10]; in a subsequent study, they also recognized both the pose and surface material of objects using diffuse reflection characteristics and data fusion [11]. Vanderschueren et al. estimated object locations using only ToF data by combining height and normal maps with an intensity map [12]. In contrast, studies integrating ToF and stereo have used reflection intensity only as a component of the depth-confidence measure, rather than as an input for recognition [22].

### 2.2. Related Work on Hand–Eye Calibration

Hand–eye calibration is a long-standing research topic in vision-based robotic systems and other systems requiring the registration of multiple coordinate systems. Various solution methods have been proposed [23,24,25,26,27], including approaches that estimate rotation and translation separately or simultaneously, and spherical targets have also been widely used as calibration targets [28,29,30]. A sphere is advantageous for the mutual registration of multiple sensors because its contour remains close to a circle regardless of the observation direction [28]. Spherical targets have also been used to estimate the extrinsic parameters of 3D sensors and LiDAR–camera systems [29,30], and retroreflective fiducial markers have been used to calibrate an industrial RGB-D camera network [31].

### 2.3. Visual Recognition in Slaughter and Meat Processing

In slaughter and meat-processing automation, visual recognition is an important component of robotic operations. Although robotic cutting and evisceration have been actively investigated, complete automation has not yet been achieved because of individual variation and the risk of tissue damage [1,2]. Specifically, Chen et al. used machine vision to recognize the positions of poultry viscera and guide grasping by an evisceration robot [3]. A related study by the same group experimentally demonstrated that the relative positions of the carcass and viscera vary significantly with individual carcass size [32]. For hygiene management, a YOLO-family detector has been applied to slaughter-line images to detect carcass contamination [4]. Because processing speed is a key constraint for line application, there is a trend toward optimizing both accuracy and speed, as seen in the case of accelerating poultry part segmentation by 1.89-fold using a lightweight network [5]. In keypoint estimation for livestock, state-of-the-art detectors, including the YOLO-Pose family, are also being evaluated for accuracy and deployability under precision livestock farming environments [33]. This study aims to meet the requirement for both accuracy and high-speed processing using a single multi-task network with inputs from a single sensor, thereby eliminating registration errors.

### 2.4. DETR-Based Object Detection

DETR [20] formulates detection as a set prediction problem and directly outputs bounding boxes with a transformer encoder–decoder without manual post-processing, such as non-maximum suppression (NMS). It is trained by mapping predictions to ground truths in a one-to-one correspondence using Hungarian matching [34]. Furthermore, the initial slow convergence issue has been substantially alleviated by deformable attention [19], which samples only a small number of locations around the reference points. RF-DETR [35] builds on the LW-DETR design [36] (a plain Vision Transformer (ViT) encoder with interleaved window and global attention, a multi-scale projector, two-stage query selection, Group DETR [37], and the intersection-over-union (IoU)-aware binary cross-entropy (IA-BCE) of [38]), replaces its encoder with a DINOv2 backbone [18], and adds weight-sharing neural architecture search, achieving real-time processing with high accuracy. The detection branch of the Proposed Network adopts the configuration of RF-DETR, which has no transformer encoder apart from the backbone (backbone, multi-scale projector, and deformable decoder); the dense keypoint path and the optional mask path are attached to the same backbone, and the boundary between the inherited and the added modules is stated in Section 3.5.

### 2.5. Self-Supervised Pre-Trained Backbone

DINOv2 [18] is a ViT backbone pre-trained through self-supervised knowledge distillation on large-scale unlabeled images, providing robust features across various downstream tasks without separate fine-tuning. In domains such as evisceration process images, where the labeled training and validation data are limited to 3250 frames acquired from nine carcasses, the quality of the pre-trained backbone is expected to be a major factor in the final performance. This study uses the public weights of RF-DETR-L (patch size 16, input resolution 704) to initialize the DINOv2-S backbone. However, because ViT outputs only single-scale (1/16) features, a separate pyramid configuration [39,40] is required for keypoint and segmentation tasks that require multi-resolution features.

### 2.6. Multi-Task Visual Recognition and Keypoint Estimation

Mask R-CNN [41] is a representative multi-task structure that integrates detection and segmentation by attaching a RoIAlign-based mask branch to a Faster R-CNN detector. YOLOv8 [16] supports detection, segmentation, and pose estimation by selectively attaching a prototype-based segmentation branch [42] and a keypoint branch on the same backbone and neck; an energy-efficient implementation on a RISC-V SoC has also been reported [43]. In the field of medical imaging, a tongue diagnosis image segmentation and multi-label classification network sharing a MobileNet backbone has shown efficiency in edge environments [17]. For keypoint estimation, RTMPose [44], which is based on the SimCC coordinate-classification representation [45], is widely deployed; however, as a top-down method, it presupposes serial coupling with a detection model. The Proposed Network eliminates this serial coupling and adopts a single-network architecture in which the keypoint queries are initialized from the box centers of the detection results and the P2 features sampled there (Section 3.9).

Single-stage keypoint estimators that do not require a separate detector have also been proposed, including the DETR-based PETR [15], ED-Pose [13], and Group Pose [46], which estimate multi-joint human poses end-to-end with pose queries, RTMO [14], a real-time one-stage method, and the YOLO-based YOLO-Pose [47] and YOLOv8-pose [16]. The Proposed Network shares with ED-Pose the idea of initializing the keypoint queries from the detection results, but it estimates only one class-specific working point per object from a single-ToF input; a quantitative comparison with the single-stage YOLOv8l-pose [16] trained on the same data is reported in Section 4.2, Section 4.3 and Section 4.4.

## 3. Materials and Methods

### 3.1. Robot System Configuration

Figure 2 shows the overall configuration of the proposed evisceration robot system. It is a dual-arm structure with two symmetrically arranged collaborative robots. Each arm consists of seven axes, including one servo axis and six UR12e (Universal Robots A/S, Odense, Denmark) axes. Since evisceration proceeds from the upper to the lower part of the carcass, there is a limit to the working range if the entire section is covered solely by the arm’s pose. To resolve this, the entire robot is designed to be vertically movable by 900 mm. Since the body length of domestic standard pigs is 1100 to 1250 mm, it is possible to track the working section of a single carcass within this range, without changing the installation position.

The ToF camera (Helios2+, LUCID Vision Labs Inc., Richmond, BC, Canada) is fixed between the two arms, and the coordinate systems of the dual-arm robot and the camera are fixed without variation. The field of view of the sensor is set so that the working point is positioned at the center of the image while considering the vertical movement. The target working distance from the camera to the carcass is 1000 mm.

The working-point output of the perception system described here is a process signal for tool-path generation and is not used as a safety function; the safeguarding of the cell and of the knife-type end-effector during automatic operation is outside the scope of this paper.

### 3.2. Luminance–Depth Image Composition

Table 1 shows the specifications of the ToF camera used in the evisceration robot system. As shown in the table, the camera outputs the x, y, and z coordinates and the luminance as 16-bit values each. Here, the input data of the learning model are generated by combining the *z*-axis depth data and the luminance. Channel 1 is a depth map that normalizes the 600–1100 mm working-area interval, within which objects are measured, to 8 bits. Channels 2 and 3 normalize the luminance to 8 bits in the intervals of 0–20,000 and 0–10,000, respectively; values outside each interval are clipped (Figure 3); these intervals were chosen for the 1000 µs exposure used for data acquisition, and a different exposure would require re-selecting them. Since the amount of reflected light changes substantially depending on the distance between the ToF camera and the object and the properties of the medium, luminance variations can be large even within a single scene. Therefore, to secure the features of both low- and high-luminance regions, the data normalized at different intervals are configured as two separate channels (the benefit of the second luminance range was not ablated; limitation (vi) in Section 5).

If the three channels are directly stacked into the R, G, and B positions (R ← C1, G ← C2, B ← C3), the format becomes identical to that of a typical 3-channel image, allowing detector architectures designed for such images to be applied without modification. Figure 3 illustrates this construction process.

### 3.3. Hand–Eye Calibration

Although the checkerboard is the de facto standard target for camera calibration [48], it is difficult to apply directly under the imaging conditions of our system because of the measurement principle of the ToF sensor. A lock-in ToF sensor calculates the distance by demodulating the reflected light at each pixel and computing its phase delay. If a single pixel spans two regions with different brightness or distance, the measured value becomes an intermediate value that corresponds to neither surface. This phenomenon, known as the mixed-pixel error, is largest at boundaries where the brightness or distance changes abruptly [8,49]. In a checkerboard, the intersections used as feature points are precisely where four black and white regions meet, i.e., where brightness changes most abruptly. Because the target pose in this work is computed from the luminance image alone (see below), the relevant consequence is that, on the low-resolution (640 × 480) active-infrared luminance image, sub-pixel corner localization at these boundaries is less reliable than the center estimation of an extended bright region; the depth error at the same boundaries would matter additionally only if the depth channel were used for target localization. The checkerboard is therefore expected to be a less favorable target for this sensor.

A second problem is the ToF camera’s own infrared illumination. In our setup, a printed grid provided only limited contrast under the infrared illumination. In addition, when the checkerboard is nearly perpendicular to the camera’s optical axis, the infrared light is reflected directly toward the camera, saturating the image, and in some poses the grid could not be recognized (in the comparison of Section 4.1, the grid target was operated at a longer exposure, 250 vs. 62.5 µs). Moreover, hand–eye calibration requires large rotational variation in the target (±30° in Section 4.1), and at large inclinations to the optical axis the grid pattern is strongly foreshortened, which further degrades corner detection at this resolution.

To address these issues, we propose retroreflective spherical markers as the calibration target. Because a retroreflective surface reflects incident light back toward the light source, it is observed as bright regardless of ambient illumination [31]. Furthermore, the feature points are obtained at the center of a bright region rather than at a contrast boundary, so the center estimate is expected to be insensitive to the symmetric blur at the boundary and to the mixed-pixel error, and the contour of the sphere remains close to a circle even with changing viewing direction [28] (strictly, the perspective image of a sphere is an ellipse whose center is slightly offset from the projected sphere center; this small systematic offset is not resolved by a repeatability experiment—limitation (vii) in Section 5). Retroreflective spherical markers have been used to achieve sub-millimeter precision in optical motion capture [50,51], although those systems rely on multi-camera triangulation at higher resolution.

Figure 4 summarizes the designs of the spherical marker target and the Radon board [52] used for comparison, along with the acquired luminance images and detection results.

As shown in Figure 2, the system uses an eye-to-hand configuration in which the ToF camera is fixed outside the robot.

Let the pose of the end-effector in the *i*-th capture be *A_i_* and the target pose measured by the camera be *B_i_*. Then, for all *i*,

*A_i_X* = *ZB_i_* (1)

holds for *i* = 1, …, *n*, where *n* is the number of captured poses. Here, the target is rigidly attached to the end-effector; *A_i_* is the transformation from the end-effector coordinate system to the robot base coordinate system (from the robot controller), *B_i_* is the transformation from the target coordinate system to the camera coordinate system measured by the camera, *X* is the transformation from the target coordinate system to the end-effector coordinate system, *Z* is the transformation from the camera coordinate system to the robot base coordinate system, and solving for *Z* is the final goal. Widely used solvers take absolute pose pairs (robot pose, camera measurement) that satisfy the eye-in-hand relation and internally build the relative-motion form *AX* = *XB*. In the eye-to-hand configuration, the pairs ($B_{i}^{-1},\;A_{i}$) are supplied to the solver in place of (*A_i_*, *B_i_*); the solver then returns *Z*^−1^, and *Z* is obtained by inversion. The detection procedure for the spherical markers is as follows; only the luminance image is used in this step. The luminance image is binarized to extract high-brightness regions, and a circle is fitted to the contour of each region to obtain its center. Since the contour of a sphere is close to a circle regardless of the observation direction, the same procedure applies regardless of the pose. Correspondences among the four markers are determined from the distance ratios between the markers, and SQPnP [53] is applied to the four corresponding points to compute *B_i_*. To exclude bias due to the solver, all five methods—Tsai–Lenz, Park–Martin, Horaud–Dornaika, Daniilidis, and Andreff–Horaud–Espiau [23,24,25,26,27]—are applied, and the result with the smallest reprojection error is adopted.

### 3.4. Dataset and Experimental Environment

Experimental data were acquired on a testbed constructed in the actual slaughter environment of the National Institute of Animal Science; example frames are shown in Figure 5. Images were captured continuously, while the robot was moving, during the evisceration of nine pig carcasses at 30 fps with an exposure time of 1000 µs, yielding 129,205 raw frames, which were converted into three-channel images as described in Section 3.2. Because consecutive frames at 30 fps are nearly identical, key frames were selected using the structural similarity index (SSIM) [54]. SSIM was computed between the most recently retained frame and each subsequent frame on the central 320 × 240 px region of the 640 × 480 image, which corresponds to the working area, and averaged over the three channels; a frame was retained and became the new reference when the averaged SSIM fell below 0.9. For reference, the SSIM between consecutive full frames had a mean of 0.9942 (maximum 0.9985), confirming that adjacent frames are near-duplicates; the threshold of 0.9 on the central region was set empirically, considering that the operation changes only part of that region. The selection retained 3823 frames, which were split at random at the frame level into 2677 training, 573 validation, and 573 held-out frames (3250 training and validation frames in total); the held-out frames were not used in this study. Because frames of the same carcass are correlated, these nine carcasses were used only for training and validation, and the test set was built from different carcasses, as described below.

The number of carcasses is set by the acquisition conditions: process images can be recorded only on the working slaughter line of the National Institute of Animal Science, on slaughter days scheduled by the facility, and each session yields a few carcasses that pass the evisceration station once. The training data therefore comprise nine carcasses with many frames per carcass.

The test set consists of five additional carcasses (p1–p5) recorded on 7 October 2026, on a day separate from the acquisition of the training carcasses (23 and 30 June 2026), on the same testbed with the same camera and acquisition settings. No frame of these carcasses was used for training or validation. The frames were converted with the same three-channel procedure, and 100 frames were taken from each carcass: the annotated frames of each carcass (about 500, obtained with the same SSIM-based key-frame selection as the training frames; 463 for p3 after 37 frames without annotations were excluded) were divided in order of acquisition time into 100 intervals, and from each interval the frame containing the most working-point classes was taken. The selection used only the annotations and was made before any network was run on these frames. This gave 500 test frames with 1334 working points (378 BUNG POINT, 257 GRASP POINT, 251 BLADDER POINT, and 448 CUT POINT). The same 500 frames were used to evaluate every network, so the comparisons between networks are paired; results for each test carcass are given in Table A1 (Appendix B). Table 2 lists the carcasses and frames in each partition, Table A3 (Appendix C) lists the acquisition date of each carcass, and examples of the test frames are shown in Figure A1 (Appendix A).

The dataset consists of frames captured at different time points during the evisceration operation. In each frame, each exposed working point is labeled with one point and one bounding box; the four classes that serve as references for robot operations are BUNG POINT, GRASP POINT, BLADDER POINT, and CUT POINT. Depending on the operation stage, some parts may not yet be exposed or may be occluded by workers, hand tools, or the robot arms and gripper, so the number of labeled classes varies from frame to frame. Annotation was performed in the Computer Vision Annotation Tool (CVAT) by a single annotator to keep the labeling consistent. In the 3823 selected frames of the nine training carcasses (of which only the 3250 training and validation frames were used in this study; Table 2), 9255 instances were labeled: 3228 BUNG POINT, 1138 GRASP POINT, 1635 BLADDER POINT, and 3254 CUT POINT; the counts differ because each working point is exposed only during part of the operation. The test frames were annotated by the same annotator with working points only; for box-based evaluation, a fixed 22 × 20 px box centered on each working point is used as the ground-truth box (Section 3.11.2).

CUT POINT is defined solely as a point, namely the incision start position, and has no object boundary. It was therefore labeled using a fixed-size box centered on the working-point coordinates. On the test set, the ground-truth boxes of all four classes are fixed 22 × 20 px boxes centered on the working points, so the box-based metrics are surrogate measures of working-point positional accuracy rather than object-boundary alignment for all four classes, and the size of the training boxes does not enter the test ground truth or the object keypoint similarity (OKS) area. The absolute AP depends on the size of this test box: Section 4.5 shows that it changes the absolute AP substantially, whereas the difference between the Proposed and Serial Networks stays within −0.20 to +0.36 AP; in the detection-AP evaluation of that section, the predicted boxes are resized as well, so those values do not depend on the box size learned from the training labels. The detection AP computed without this resizing (Section 4.2) differs from the 22 × 20 px detection-AP evaluation of Section 4.5 by at most 0.06 AP for every network, so this learned box size has a negligible effect on the reported detection AP.

Because each working point is directly used to generate the robot’s tool path, pixel-level positional accuracy is essential.

### 3.5. Overall Architecture

The overall configuration of the proposed architecture is shown in Figure 6. The 704 × 704 input image passes through the DINOv2-Small backbone (patch 16, dimension 384, depth 12), and the intermediate features of blocks 3/6/9/12 (each with 384 channels, 44 × 44) branch into the following paths.

(1) Detection path—the MSP fuses the block 3/6/9/12 features to generate a single-scale feature P4 (256 channels, 44 × 44), and the deformable DETR decoder outputs the bounding box, class, and layer-wise query feature hs.
(2) Dense prediction path—KPT·Seg FPN maps the block 3/6/9/12 features to a multi-resolution pyramid P2/P3/P4/P5 (256 channels each, 176^2^/88^2^/44^2^/22^2^). P2 is refined once more through a UNet structure [55] to become P2′ (176 × 176).
(3) Keypoint path—the keypoint query is initialized with the center coordinates of the detected bounding box and the P2 feature sampled at that location. The class-specific working-point coordinates are refined using a deformable decoder that references the FPN levels P2/P3/P4 (distinct from the MSP output P4 of the detection path).
(4) Instance segmentation path (optional)—generates query-specific masks through the dot product of P2′ and the query feature hs. This is activated only for tasks with mask annotations.

The total number of parameters is 43.9 M. The detection branch (backbone, projector, and decoder, together with the detection losses) is that of RF-DETR-L (33.6 M); the KPT·Seg FPN, the UNet refinement, the keypoint decoder, and the optional mask head add 10.3 M. All tasks operate through decoders without a separate transformer encoder.

### 3.6. DINOv2-Small Backbone

The backbone is the DINOv2-S variant distributed with RF-DETR [35]: patch size 16 (the original DINOv2 uses 14 [18]), register tokens [56], and the interleaved windowed/global attention of LW-DETR [36], which suppresses the computational cost even for high-resolution inputs. The pre-trained weights are extracted from RF-DETR-L; because they have been adapted to the Common Objects in Context (COCO) detection task, we expect faster convergence in detection-related downstream tasks than with pure DINOv2 weights. Among the 12 layers, the outputs of blocks 3, 6, 9, and 12 are extracted as intermediate features, all with 384 channels and a resolution of 44 × 44 for a 704 × 704 input. Because the shallow layer (block 3) contains low-level information such as boundaries and textures, whereas the deep layer (block 12) contains semantic information, these intermediate features provide the downstream fusion modules with complementary information at different levels of abstraction.

### 3.7. MSP and Deformable DETR Decoder

Figure 7 shows the structure of the MSP. The MSP concatenates the four features from different depths along the channel axis (all-to-all concat, 1536 channels), projects them to 256 channels using a 1 × 1 convolution, and then refines them with three C2f blocks [16], as in LW-DETR [36], to obtain P4. Because ViT lacks hierarchical downsampling and all blocks have the same spatial resolution, this structure combines semantic diversity across layer depths rather than spatial scales. The information from all four depths is fused by concatenation.

The number of decoder queries is 300. In deployment, however, only the highest-scoring query per class, i.e., four queries in total, is passed to the keypoint head, and a class whose top-1 score falls below the operating score threshold is reported as absent; the increased number of queries therefore affects only the decoder self-attention cost and does not affect the latency of the subsequent stage. In the evaluation, keypoints were decoded for every query with a score of at least 0.01, so that the AP and the outcomes at the operating threshold cover every prediction; in the forced-N latency test, they were decoded for the N highest-scoring queries (Section 3.11.2).

The decoder is a 4-layer deformable DETR decoder, and the deformable cross-attention operation in each layer is shown in Figure 8. Class scores and boxes are predicted for all locations of the single-scale P4, and the top 300 predictions are selected as the initial reference boxes through two-stage query selection. Each layer predicts a box correction and passes the refined box to the next layer as the reference box for box refinement. Group DETR [37] (13 groups) is used during training. Each layer consists of self-attention (8 heads), deformable cross-attention (16 heads, 2 sampling locations per head), and a feed-forward network (FFN; 2048 dimensions).

As shown in Figure 8, each query predicts the offsets Δp and weights A through a linear projection, samples the P4 features at locations displaced by Δp from the center of the reference box (cx, cy, w, h), and computes their weighted sum using A. The offsets Δp are scaled in proportion to the box size, and A is softmax-normalized for each head. Because the sampling and aggregation cost per query is proportional to the number of heads, levels, and sampling points rather than the number of feature-map locations, only 32 sampling locations (16 × 1 × 2) are sampled per query; the value projection is still computed over the full 44 × 44 map, but it is shared by all queries. The decoder outputs class logits and boxes (cx, cy, w, h) at each layer, and the box centers of the queries selected at the final layer are used to initialize the queries of the keypoint branch.

### 3.8. KPT·Seg FPN and Instance Segmentation

Figure 9 shows the structure of the KPT·Seg FPN. To compensate for the single-scale limitation of ViT, the KPT·Seg FPN generates P2 (176^2^), P3 (88^2^), P4 (44^2^), and P5 (22^2^) from the features of blocks 3/6/9/12 using two 2× deconvolution layers, one 2× deconvolution layer, a 1 × 1 projection, and a stride-2 convolution, respectively; this follows the simple feature pyramid used for plain ViT backbones [39], except that the four levels are built from four different depths (blocks 3/6/9/12) rather than from the last block alone, in the spirit of the multi-depth reassembly of DPT [40], so that the keypoint head receives features of different semantic depth at each resolution. P2/P3/P4 are passed to the keypoint head without fusion, whereas P5 is used only as the starting point of the UNet. Because features from different depths are used, the lack of feature diversity in a pyramid that copies the same feature while only changing its resolution is expected to be avoided, and because no fusion operation is performed, little latency is added; the contribution of this choice was not isolated (limitation (vi) in Section 5). Since tissue boundaries and keypoints around the working points are often distinguished by subtle changes in texture and intensity, the quality of the high-resolution, low-level feature (P2) is expected to affect keypoint precision. The subsequent three-stage UNet cascade starts from P5 and repeatedly performs 2× deconv upsampling and 3 × 3 convolution with channel layer normalization and the Gaussian error linear unit (GELU), incorporating P4, P3, and P2 at the corresponding resolutions through skip connections at each stage to produce the final P2′ (256 ch, 176 × 176).

In tasks requiring instance segmentation, as shown in Figure 10, the mask logit for each query is the dot product of the projected UNet-refined P2′ ∈ ℝ 256 × 176 × 176 (a depthwise-convolution block followed by a 1 × 1 convolution) and the projected decoder query feature hs (a multilayer perceptron (MLP) followed by a linear layer), and the mask probability is its sigmoid

(2) $$M_{n}(u,v)=\sigma \left(\sum_{c=1}^{256}hs_{n,c}\cdot P{2^{\prime }}_{c}(u,v)\right).$$

In Equation (2), *n* is the query index, (*u*,*v*) represents the pixel coordinates, σ is the sigmoid function, and *hs*_n_ and P2′ denote the values after projection.

Although this approach is similar to the prototype combination used in YOLACT [42], the detection query feature is projected by a small MLP and used as the coefficients instead of using a separate coefficient prediction branch. Consequently, the additional parameters amount to only approximately 0.9 M. In this evisceration task, the segmentation branch was deactivated because no mask annotations are available.

### 3.9. Keypoint Branch

The keypoint head utilizes the detection result as the initial query value (Figure 11). It projects the center coordinates (cx, cy) of the detected object into a coordinate embedding, and initializes the keypoint query by adding the features obtained by bilinear sampling at the corresponding location in P2

*q_k_* = *proj_xy_*(*c_x_*,*c_y_*) + *proj_feat_*(*sample*(*P*2, (*c_x_*,*c_y_*))). (3)

In Equation (3), *q_k_* is the initial query of the *k*-th detected object (one per class in deployment; Section 3.7), (*c_x_*, *c_y_*) is its box center, proj_xy_(·) is the coordinate-embedding mapping, sample(P2, ·) denotes bilinear sampling of P2 at the box center, and proj_feat_(·) is the projection of the sampled feature. Coordinate embeddings provide the positional prior and sampling features provide the appearance information of the corresponding object.

The initialized query passes through a 3-layer deformable decoder (8 heads, 8 sampling points per head on each of the three levels) that references the three scales P2/P3/P4 (Figure 11). The first layer determines the initial coordinates, while the subsequent layers refine these coordinates by cumulatively predicting their offsets from the initial coordinates.

The error is not directly regressed as a single value; instead, the probabilities of 17 displacement candidates in each of x and y are obtained via softmax, and the weighted average Σ_j_ p_j_·v_j_ is computed (an integral, i.e., soft-argmax, coordinate representation over a discrete distribution, as used for box edges in Generalized Focal Loss (GFL) [57] and labeled “17-bin integral” in Figure 6; the distribution focal loss (DFL) itself is not used). The candidate intervals are narrow near 0 and wider farther away (up to ±4, divided by a refine scale of 4 to give ±1 in normalized coordinates), a spacing intended to handle both large displacements and fine adjustments (not ablated; limitation (vi) in Section 5), and the concentration of the probability distribution could serve as a confidence indicator (not used in this work).

During training, convergence is accelerated by concurrently using a denoising query [58] that reconstructs the original coordinates from ground-truth coordinates with added noise.

### 3.10. Loss Function

The training loss is the sum of the detection, keypoint, and instance segmentation terms, *L* = *L*_det_ + *L*_kpt_ + *L*_seg_, with *L*_seg_ = 0 in this work.

#### 3.10.1. Detection Loss

Correspondence between the predicted queries and ground truths is determined using Hungarian matching [34]. The detection loss is a weighted sum of the classification loss and the L1 and generalized IoU (GIoU) [59] losses for bounding-box regression

ℒ*_det_* = *λ_cls_*ℒ*_cls_* + *λ_L_*_1_ℒ*_L_*_1_ + *λ_giou_*ℒ*_GIoU_*. (4)

In Equation (4), *λ_cls_* = 1, *λ_L_*_1_ = 5, and *λ_giou_* = 2. Auxiliary supervision is used to apply the same loss to all decoder layers and the two-stage initial proposal.

For the classification term *L*_cls_, the IoU-aware BCE (IA-BCE) of [36,38], inherited unchanged from RF-DETR-L, is used instead of the standard focal loss [60]; IA-BCE reuses the focal-loss constants α = 0.25 and γ = 2 as follows. When the target value of a matched query is always set to 1, the classification score saturates and fails to reflect the box quality. IA-BCE sets the target score of a matched query to *t* = *p*^α^ · IoU^(1−α)^ (exponent α = 0.25), thereby linking it to the IoU of the predicted box, and applies a weight of *p*^γ^ (γ = 2) to unmatched queries. This allows the classification score to serve as a proxy for localization quality. Because the baseline RF-DETR also uses the same classification loss, the classification-loss conditions are identical for both models. The use of IA-BCE is confined to the loss function; therefore, the inference path and parameter count remain unchanged.

#### 3.10.2. Keypoint Loss

The coordinate L1 loss and the OKS-based loss [47] are used

ℒ*_kpt_* = *λ*_1_ℒ*_L_*_1_ + *λ*_2_ℒ*_OKS_*. (5)

In Equation (5), *λ*_1_ = 10 and *λ*_2_ = 4, where *L*_L1_ here is the L1 loss of the normalized keypoint coordinates and *L*_OKS_ = 1 − OKS [47]. L1 provides direct regression of coordinates, while the OKS term provides scale-invariant supervision aligned with the evaluation metric. The same loss configuration is also applied to the denoising query.

#### 3.10.3. Instance Segmentation Loss (Optional Activation)

When the segmentation branch is activated, the sum of binary cross-entropy and Dice loss [61] is used for the matched query–ground truth pairs

(6) $$L_{\mathrm{seg}}=L_{\mathrm{BCE}}+L_{\mathrm{Dice}},\;L_{\mathrm{Dice}}=1-\frac{2|\hat{Y}\cap Y|}{\left|\hat{Y}|+|Y\right|}.$$

In images dominated by background pixels, the Dice term compensates for the class imbalance that occurs when only BCE is used.

### 3.11. Training Setup and Evaluation Metrics

#### 3.11.1. Training Setup

All networks were trained on the same training and validation frames and repeated with three random seeds (42, 43, and 44); results are reported as the mean ± sample standard deviation over the three runs. The detection branch of the Proposed Network uses the official RF-DETR-L implementation (rfdetr 1.9.1) and is initialized from the RF-DETR-L COCO checkpoint for all layers; it is trained with the same hyperparameters as the detector of the Serial Network (batch size of 32, learning rate of 1.3 × 10^−4^). No data augmentation was applied to either detector: the augmentation pipeline of rfdetr generated no augmentations with the installed version of the albumentations library, and the serial detector was trained under the same condition. The keypoint OKS loss uses σ = 0.079 and the area of the annotated box. The Serial Network uses an RF-DETR-L detector trained with these settings (rfdetr 1.8.0.dev0) followed by four class-wise RTMPose-M models [44], and its batched variant uses the same detector followed by one four-point RTMPose-M model that processes all crops of a frame in a single call. YOLOv8l-pose [16] was trained on the same split. The RTMPose-M models were trained for 200 epochs on 256 × 256 crops of the detected boxes with the default MMPose augmentation (rotation, scaling, color, blur, and dropout), starting from a CSPNeXt-m backbone pre-trained on AI Challenger (AIC) and COCO; YOLOv8l-pose was trained for 200 epochs at an input size of 704 with the default Ultralytics augmentation (mosaic, horizontal flip, color, and scaling), starting from the COCO-pose checkpoint. These settings differ from those of the Proposed Network, which was trained without data augmentation, and no matched hyperparameter search is reported; the comparison therefore reflects each method as trained here rather than its best achievable accuracy (Section 5).

#### 3.11.2. Evaluation Metrics

Following the COCO protocol [62], detection is evaluated by the AP over IoU thresholds of 0.50–0.95 and keypoints by the AP over OKS thresholds of 0.50–0.95 (interval of 0.05), together with AP50 and AP75. The OKS uses a single constant σ = 0.079 for all four working-point classes and the area of the ground-truth box as the object area (OKS = exp(−d^2^/(2s^2^k^2^)), where d is the distance between the predicted and ground-truth working points, s^2^ is this area, and k = 2σ, as in the COCO keypoint evaluation); with the fixed 22 × 20 px test boxes, an OKS of 0.5 corresponds to a distance of 3.90 px for every class. Class-specific constants were not estimated, because this requires repeated annotations of the same points by several annotators. The sensitivity of the results to the box size and σ is reported in Section 4.5.

Working-point errors are computed for every ground-truth point: each point is paired with the top-scoring prediction of its class, without a score threshold, so that points missed at the operating threshold are included in the error statistics rather than excluded. The mean, median, and 95th- and 99th-percentile errors are reported per class. Operational outcomes are evaluated at the operating threshold of the robot software (detection score > 0.3). For every network, every prediction above this score is taken as an output, so that a class can yield more than one output per frame and missed detections, duplicates, and spurious working points are all counted. This counting rule is broader than the deployed output rule (Section 3.7), which keeps only the highest-scoring output of each class; the outcomes under the deployed rule were not tabulated separately, so the rates reported in Section 4.4 describe all outputs above the threshold rather than only the outputs passed to tool-path generation. The outputs above the threshold are matched to the ground truth with the standard detection confusion matrix (IoU 0.45, class-agnostic matching), and a matched prediction of the correct class is counted as a correct working point when its OKS is at least 0.5 and as a mislocalized working point otherwise. Precision, recall (correct working points divided by all ground-truth points; a ground-truth point matched by a mislocalized prediction counts neither as recalled nor as missed), the miss rate (unmatched ground-truth points divided by all ground-truth points), and the false-working-point rate (outputs that are not correct working points divided by all outputs) are reported per class.

To compare the working-point errors of two networks, the difference in their mean errors over the same 1334 ground-truth points is computed for each training seed, and its 90% confidence interval is estimated with a carcass-level bootstrap (the five test carcasses resampled with replacement 2000 times), so that correlated frames of the same carcass are not treated as independent. A margin of 0.5 px (about 1 mm at the 1000 mm working distance) is used to summarize these intervals; this margin was set after the analysis and is not derived from a tool tolerance.

The comparison targets are a serial detector and pose-estimator pipeline (Serial Network), in which an RF-DETR-L detector is followed by class-wise RTMPose-M pose estimators that process the detected crops one at a time; its batched variant (Serial Network (batched)), in which the same detector is followed by one four-point RTMPose-M model that processes all crops of a frame in a single call; and the single-stage keypoint estimator YOLOv8l-pose [16]. The proposed single network is denoted as the Proposed Network.

Latency is measured on a single GeForce RTX 5090 graphics processing unit (GPU; NVIDIA Corporation, Santa Clara, CA, USA) with a batch size of 1, for PyTorch 32-bit floating-point (FP32) and TensorRT FP16 (PyTorch 2.9.1 with CUDA 12.8 and TensorRT 10.15.1.29; YOLOv8l-pose was run with PyTorch 2.11.0 and Ultralytics 8.4.142 in a separate environment). For each condition, the first 70 test frames are used: 30 warm-up frames followed by 4 rounds of the same 40 frames (160 timings), with the conditions interleaved and CUDA synchronization before each timer read. The measured scope is the network stage, from the resized input tensor to the working points in image coordinates: the common resize and normalization to 704 × 704 is excluded, whereas the crop pre-processing of the serial pipelines and the coordinate post-processing are included. Image acquisition, depth-based 3D conversion, hand–eye transformation, and robot-coordinate output are not included. Latency is reported on the deployed path (for the Proposed Network, the highest-scoring query of each of the four classes passed to the keypoint head, as in Section 3.7; for the other networks, the objects detected in each test frame) and with the number of objects per frame N forced to 1, 2, 4, 8, and 16 (N = 8 and 16 exceed the four working-point classes of this task and serve as a scaling test), as the mean ± standard deviation (SD) over the three trained seeds (this SD reflects differences between trained models); frame-to-frame variation is summarized by the 99th-percentile latency on the deployed path. In the forced-N test, the Serial Network passed the N highest-scoring of its 300 RF-DETR-L detections to the class-wise RTMPose-M models in N calls, and its batched variant passed them to the four-point RTMPose-M model in one call with a batch of N; the Proposed Network passed the N highest-scoring of its 300 detection queries to the keypoint head in one call; and for YOLOv8l-pose, which outputs boxes and keypoints together, the confidence threshold was lowered to 10^−6^ and the N highest-scoring detections after non-maximum suppression were kept.

#### 3.11.3. TensorRT FP16 Deployment

For field deployment, the trained models were converted into TensorRT FP16 engines. The models were exported from PyTorch to the Open Neural Network Exchange (ONNX) format and then converted to TensorRT, with the sampling operation of deformable attention exported as the standard ONNX operator grid_sample. All four networks were converted in the same way, with only the FP16 flag set and no layer of any network explicitly kept in FP32. The accuracy and working-point errors of the engines are evaluated on the same 500 test frames (Section 4.3).

## 4. Results

### 4.1. Hand–Eye Calibration Repeatability

The repeatability of the complete calibration procedure built on the proposed retroreflective spherical marker was compared with that of a procedure built on a grid target; because the target, its detector, and the exposure time change together, the comparison is between the two complete procedures rather than between the targets alone. The two targets are shown in Figure 4a. For the spherical target, four retroreflective spheres with a diameter of 30 mm were fixed to a rigid frame, and coordinates of (0, 0), (70, 60), (140, −90), and (280, 0) mm were assigned (with positive x to the right and positive y upward). As the grid target, a Radon board with a size of 240 mm × 200 mm was used, which has 5 × 4 internal intersection points at 40 mm intervals. Because the ToF camera provides only three selectable exposure times—1000, 250, and 62.5 µs—the exposure time was set to 62.5 µs for the spherical marker and 250 µs for the Radon board, while all other settings were kept identical. The spherical markers were detected using the method described in Section 3.3, and a local Radon transform-based detector [52] was used to detect the grid intersection points. Figure 4b shows the captured images, and Figure 4c shows the detection results.

The robot, camera, and working volume were kept fixed, and only the target, its detector, and the exposure time appropriate to each target (62.5 vs. 250 µs) were changed. For each target, 150 poses were automatically captured by randomly sampling positions within the working volume and applying rotations of ±30°. This procedure was repeated five times.

Figure 12 shows the convergence of the hand–eye calibration solution as a function of the number of data points *n*. In (a), the deviation is defined as the distance (mm) between the camera position obtained by re-solving the calibration using only *n* poses from a single experiment and a reference point. The reference point is the mean camera position of the solutions obtained using the complete data from all five experiments. Because the reference point is the mean across the five experiments, the curves converge not to zero but to a level corresponding to the inter-experiment scatter; this deviation is therefore a measure of repeatability, not of absolute accuracy. Panel (b) shows the residuals of the same solutions, with the shaded regions indicating the difference between the two targets.

The spherical marker converges faster initially: its deviation falls below 3 mm at *n* ≈ 10, whereas that of the Radon board remains at the 8 mm level. This difference persists as *n* increases. At *n* = 30, 90, and 150, the deviation of the Radon board was 2.7, 3.4, and 3.1 times that of the spherical marker, respectively, and at *n* = 150 the final deviations were 0.374 mm (spherical marker) and 1.157 mm (Radon board). The residuals also remained different by approximately 0.8 mm, at 0.56 and 1.34 mm, respectively. Because the robot, camera, and working volume were identical, this difference is attributable to the target–detector–exposure combination rather than to the robot or the camera.

The rotational component was evaluated from the same five solutions per procedure. The rotation of each solution was compared with the mean rotation of the five on SO(3), and the geodesic angles were summarized as an RMS: the rotational repeatability was 0.022° for the procedure built on the spherical marker and 0.073° for that built on the Radon board (3.3 times), and the largest difference between any two calibrations was 0.049° and 0.163°, respectively. To express both components at the working points, each of the five solutions was applied to points spanning the camera field of view at 600, 1000, and 1100 mm, and the RMS displacement from the mean solution was computed; because the full pose (rotation and translation) of each solution was applied jointly, the two components were not added independently. At the 1000 mm working distance, this displacement was 0.30–0.43 mm for the spherical-marker procedure and 0.65–1.76 mm for the Radon-board procedure (0.28–0.46 and 0.64–1.85 mm over 600–1100 mm). Like the translational deviations, these values measure repeatability; absolute accuracy against an independent reference was not evaluated.

### 4.2. Accuracy on Carcasses Not Used for Training

Table 3 shows the COCO summary metrics on the 500 test frames of the five carcasses not used for training (mean ± SD over three training runs). The Proposed Network reached a detection AP of 68.63 ± 0.27 and a keypoint AP of 73.27 ± 0.36, compared with 68.28 ± 0.27 and 73.17 ± 0.11 for the Serial Network. The batched Serial Network uses the same detector and therefore gave the same detection results, and YOLOv8l-pose reached 68.27 ± 0.22 and 73.84 ± 0.21. The Proposed Network and the Serial Network differed by less than 1 AP in all six metrics (largest difference +0.80, keypoint AP75); across all four networks, the largest spread was 2.08 (keypoint AP75).

Figure 13a and Table 4 compare the working-point errors by class over all 1334 ground-truth points at the original resolution of 640 × 480. The overall mean error was 1.83 ± 0.12 px for the Proposed Network, 2.01 ± 0.05 px for the Serial Network, 2.01 ± 0.04 px for the batched Serial Network, and 2.45 ± 0.69 px for YOLOv8l-pose. The mean error of the Proposed Network was lower than that of the Serial Network in all four classes, by 0.03 px (GRASP POINT) to 0.34 px (BUNG POINT), differences comparable to the variation across training runs; YOLOv8l-pose varied more between training runs, mainly because of one seed (Table 5).

Table 5 reports the paired comparison with the Serial Network for each training seed. The difference in mean error between the Proposed Network and the Serial Network was −0.08, −0.31, and −0.14 px for the three seeds (negative values indicate a smaller error for the Proposed Network), and the upper bound of the 90% carcass-level bootstrap interval was below +0.5 px for all three. The batched Serial Network gave the same errors as the Serial Network (differences −0.01 to +0.01 px), whereas for YOLOv8l-pose the upper bound was below +0.5 px for one of the three seeds. Relative to the labels of a single annotator, the working-point error of the Proposed Network is therefore comparable to that of the Serial Network (Table 5). Because one pixel of the ToF camera corresponds to approximately 2 mm of lateral displacement at the 1000 mm working distance (about 2.3 mm at the far end of the 600–1100 mm range), a difference of 0.08–0.31 px is a nominal lateral displacement of about 0.2–0.6 mm; the accuracy of the 3D working point in the robot frame, which additionally depends on depth and calibration errors, is not evaluated here (Section 5), whereas related cutting-point work has evaluated such accuracy directly in 3D [63]. Results for each test carcass and the class-wise AP are given in Table A1 and Table A2 (Appendix B); carcass p4 was the most difficult for all networks (keypoint AP 59.66–62.21).

Figure 14 shows, for one test frame per class (seed 42; frames chosen for illustration), the output distributions from which each network computes the working-point coordinate. The Proposed Network displays the raw 17-bin integral distributions (for each of x and y) from the final layer of the keypoint head, within a window of ±0.07 in normalized coordinates around the distribution center; the two Serial Networks display the raw SimCC output distributions of RTMPose-M, computed from the region cropped with the detector’s predicted box to reproduce the actual inference conditions. YOLOv8l-pose has no coordinate distribution; its column shows the density of the class keypoints of all anchors before non-maximum suppression, weighted by the class score, and therefore has a different meaning. Because the distributions have different bin resolutions, the comparison focuses on the locations of the concentrated activations rather than on their shapes. For all four classes, the peak of the distribution of the Proposed Network lies at or near the ground-truth working point, and the activation regions are concentrated on the same anatomical regions. The distribution of the Serial Network appears sharper; contributing factors are the upsampling of an object a few tens of pixels across to the crop resolution and the finer bin resolution, which are not separable here, and the upsampling is obtained at the cost of additional latency per object.

Although the distribution of the Proposed Network is wider at the original scale, the working point is computed from its expectation rather than its peak, and the resulting pixel error is comparable to that of the Serial Network (Figure 13a).

### 4.3. Inference Latency and TensorRT FP16 Deployment

Table 6 and Figure 15 and Figure 16 report the network-stage latency of the four networks. With the TensorRT FP16 engines used for deployment, the latency on the deployed path was 2.34 ms for the Proposed Network, 4.11 ms for the Serial Network, 3.40 ms for the batched Serial Network, and 2.23 ms for YOLOv8l-pose; the 99th-percentile latencies on the deployed path were 2.52, 5.83, and 4.25 ms for the first three (the per-frame timings recorded for YOLOv8l-pose, from which the percentile is computed, include the pre-processing of the Ultralytics implementation, so its 99th percentile is not reported). When the number of objects per frame was forced from 1 to 16, the latency of the Proposed Network stayed between 2.05 and 2.37 ms, whereas that of the Serial Network grew from 2.73 to 17.22 ms and that of the batched Serial Network from 2.81 to 9.44 ms; batching therefore reduced, but did not remove, the growth of the serial latency with the number of objects. At N = 4, the number of working-point classes, the latency of the Proposed Network was 0.41 times that of the Serial Network and 0.58 times that of the batched Serial Network. The single-stage YOLOv8l-pose had a network-stage latency similar to that of the Proposed Network (2.23–2.27 ms), so the latency advantage of the Proposed Network is stated relative to the two-stage pipeline it replaces. Measured stage by stage with TensorRT FP16 on the deployed path, the pre-processing stage took 3.80 ms for the Proposed Network and 4.77 and 4.78 ms for the Serial and batched Serial Networks; it consists of the resize and normalization to 704 × 704, which is excluded from Table 6, plus, for the serial pipelines, the crop pre-processing, which is included; the pre-processing cost depends on the implementation (the pre-processing stage of the Ultralytics implementation of YOLOv8l-pose, which performs its own resizing and is likewise excluded from Table 6, took 0.62 ms). The inference stage took 2.19, 2.82, and 2.14 ms, and the post-processing stage took 0.18, 0.37, and 0.38 ms, for the Proposed, Serial, and batched Serial Networks, respectively. Including the pre-processing as implemented here, the per-frame time of YOLOv8l-pose (0.62 + 2.23 ms) was shorter than that of the Proposed Network (3.80 + 2.34 ms).

In PyTorch FP32, the ranking differed (Figure 15 and Table 6): on the deployed path the batched Serial Network (14.74 ms) was faster than the Proposed Network (17.87 ms), and it was slower only with 16 objects per frame (19.90 vs. 17.99 ms), while the sequential Serial Network took 22.92 ms on the deployed path and 89.19 ms at N = 16. The speed comparison is therefore stated for the TensorRT FP16 engines used for deployment (Figure 16). In all TensorRT FP16 conditions, all networks remained within the 33.3 ms frame interval of the 30 fps camera, so the practical benefit of the remaining difference is a latency that does not grow with the number of objects and the GPU time it may leave for other process recognition tasks, rather than the ability to keep up with the camera.

Table 7 reports the accuracy of the same TensorRT FP16 engines on the 500 test frames. For the Proposed Network, the conversion changed the detection AP by −0.10 and the keypoint AP by −0.20, and the mean and median working-point errors were 1.83 and 1.07 px, compared with 1.83 and 1.06 px in PyTorch FP32. For all four networks, the conversion changed the AP by at most 0.3 points and the mean working-point error by at most 0.03 px; within each class, the mean error of the Proposed Network and of both Serial Networks changed by at most 0.03 px (Figure 13b), so the working-point precision of these three networks is preserved in the engines that provide the reported latencies; for YOLOv8l-pose, the class-wise mean errors changed by up to 0.33 px.

### 4.4. Failure Analysis at the Operating Threshold

Table 8 reports the working-point outcomes at the operating threshold (detection score > 0.3). No prediction was assigned to a different working-point class for any network or seed, so the failures consisted only of missed detections and mislocalized or spurious working points. Over all classes, the Proposed Network had a precision of 93.75 ± 0.38%, a recall of 92.25 ± 0.30%, a miss rate of 3.15 ± 0.07%, and a false-working-point rate of 6.25 ± 0.38%, compared with 93.84 ± 0.27%, 92.55 ± 0.50%, 2.87 ± 0.41%, and 6.16 ± 0.27% for the Serial Network. The remaining ground-truth points were matched by a prediction of the correct class with an OKS below 0.5, that is, an error above 3.90 px; this mislocalized-point rate (100% − recall − miss rate) was 4.60% for the Proposed Network and 4.58% for the Serial Network overall, and 8.17% and 7.91% at GRASP POINT, so mislocalization, rather than missed detection, was the more frequent failure over all classes at the operating threshold. GRASP POINT was the most difficult class for all networks (recall 86.90–87.55%, false-working-point rate 10.47–12.07%). Table 9 gives the per-class error percentiles over all ground-truth points: the medians were 0.64–1.37 px and the 95th percentiles 3.68–7.06 px, whereas the 99th percentiles revealed heavy tails for some classes, e.g., 28.41 ± 8.87 px for the Serial Network and 17.77 ± 7.91 px for the Proposed Network at BLADDER POINT; at GRASP POINT and CUT POINT, the Proposed Network had higher 95th and 99th percentiles than the Serial Network (GRASP POINT: 6.13 vs. 5.36 and 16.65 vs. 14.92 px; CUT POINT: 4.46 vs. 4.32 and 10.08 vs. 9.47 px).

### 4.5. Sensitivity to the OKS Definition

Table 10 re-scores the same predictions with the OKS area replaced by 16 × 16, 32 × 32, and 50 × 50 px boxes, with σ = 0.025, 0.05, and 0.107, and, for the detection AP, with the ground-truth and predicted boxes both resized to these sizes. The absolute AP changed substantially with the definition (keypoint AP 25.66–93.72, detection AP 60.08–87.10), whereas the difference between the Proposed Network and the Serial Network remained within −0.20 to +0.36 AP in all conditions. The ranking of the networks changed with the definition, so the differences between the networks are smaller than the effect of the evaluation definition, and the conclusion that the Proposed and Serial Networks perform comparably did not change over the box sizes (16 × 16 to 50 × 50 px) and σ values (0.025 to 0.107) tested.

## 5. Discussion

By synthesizing the experimental results, the practical significance of the proposed system can be summarized into three aspects.

First, because the color camera is excluded and the input is composed of only the depth and luminance obtained from the same pixel of the same optical system, registration errors between images, which inevitably occur in RGB-D configurations, do not exist, and the geometric relationship between the robot and the camera is also estimated from the same luminance image. Under this condition, the choice of calibration target, its detector, and the exposure setting determined the repeatability. The calibration procedure built on the retroreflective spherical marker showed 3.1 and 3.3 times better translational and rotational repeatability, respectively, than the grid-based procedure (0.374 vs. 1.157 mm and 0.022° vs. 0.073° at *n* = 150); for the marker-based procedure, this corresponds to a working-point repeatability of 0.30–0.43 mm at the 1000 mm working distance (Section 4.1).

Second, the single-network configuration removes the second model and the crop-and-resize stage of the serial pipeline. With the TensorRT FP16 engines, its network-stage latency on the deployed path was 0.57 times that of the Serial Network and 0.69 times that of the batched Serial Network, and it stayed nearly constant as the number of objects increased, whereas the latency of both serial variants grew with it (Section 4.3). Whether the structure in which the keypoint queries are initialized from the detection outputs within the same network also changes how upstream detection errors propagate downstream (the first drawback in Section 1.3) was not examined here. Relative to the sequential Serial Network, the reduction comes from executing one backbone pass and one keypoint-decoder pass for all objects per frame instead of one detector pass plus N pose passes, and from removing the crop pre-processing; relative to the batched Serial Network, whose inference stage took about as long as that of the Proposed Network (2.14 vs. 2.19 ms), it comes mainly from removing the crop pre-processing (Section 4.3), so this part of the advantage applies to the crop implementation measured here; the accuracy–latency trade-off is attributed to this single-pass, shared-backbone design as a whole, and its individual design choices were not ablated (limitation (vi) below). A single-stage estimator (YOLOv8l-pose) reached a similar network-stage latency, so the latency advantage is specific to replacing the two-stage pipeline; including the pre-processing as implemented here, YOLOv8l-pose was faster per frame (Section 4.3).

Third, on five carcasses not used for training, the speedup is achieved at a comparable working-point error. The mean working-point error was 1.83 vs. 2.01 px (Proposed vs. Serial), the paired differences were −0.08 to −0.31 px with 90% upper bounds below +0.5 px for all three training runs, and the COCO metrics were within about 1 AP of each other (Section 4.2); the miss and false-working-point rates at the operating threshold were also similar (Section 4.4). Relative to YOLOv8l-pose, the Proposed Network had a similar keypoint AP (73.27 vs. 73.84) and a lower mean working-point error (1.83 ± 0.12 vs. 2.45 ± 0.69 px), mainly because of one of the three YOLOv8l-pose runs (Table 5); the median errors were similar (1.06 vs. 1.03 px, Table 7). Because all deployed engines are already below the 33.3 ms frame interval, the practical value of the shorter, constant network latency lies in the computational resources it may free for other process recognition tasks on the same GPU. A short network latency is a necessary but not a sufficient condition for continuous robotic operation, which also depends on the coordination of perception, motion, and the process sequence [64]; a closed-loop trial is outside the scope of this paper.

Finally, the limitations of this study fall into three groups: the evaluation scope, the baselines and ablations, and the sensor-side evidence. Evaluation scope: (i) The training data come from nine carcasses at one facility, and the five test carcasses were recorded on the same testbed with the same camera and settings; repeated leave-one-carcass-out testing was not performed. The 100 frames of each test carcass are a subset of its recording, so the class counts of the test set (Section 3.4) depend on the frames included and do not represent the whole operation of each carcass; because the frame with the most working-point classes was taken from each interval, heavily occluded frames are likely under-represented. Data from other slaughterhouses and line conditions are required to establish generalization beyond this facility. The frames were acquired on the testbed while the robot was moving, so the occluders in the data include the robot’s arms and gripper as well as operators’ hands and hand tools (Figure 5, Figure A1 and Figure A2); no closed-loop trial in which the working points output by this network drove the robot is reported here. (ii) Results are reported as the mean ± SD over three training runs, and the confidence intervals of Section 4.2 resample carcasses rather than frames; with five test carcasses, these intervals are approximate, and the 0.5 px margin used to summarize them was set after the analysis. (iii) All annotations were produced by a single annotator, so annotator variability was not measured, class-specific OKS constants could not be estimated, and the comparison is relative to the labels of this annotator; the classes are also imbalanced (1138 GRASP POINT vs. 3254 CUT POINT instances in the 3823 selected frames). (iv) The miss and false-working-point rates are reported at the operating threshold with every prediction above it counted (Section 4.4), not for the per-class output of the deployed rule (Section 3.7); the behavior of the robot on a missed or mislocalized working point is not evaluated. Baselines and ablations: (v) The comparison covers one serial detector and pose-estimator pipeline, its batched variant, and one single-stage baseline (YOLOv8l-pose) trained on the same data, with the RTMPose-M models and YOLOv8l-pose trained under settings not matched to those of the Proposed Network and no matched hyperparameter search reported; RTMO [14] and ED-Pose [13] have not been compared. (vi) Design choices such as the decoder-only detection head, single-point keypoint query initialization from the detection outputs, the integral (soft-argmax) keypoint head, the number of sampling points in the keypoint decoder, the omission of a per-object feature encoder (region-of-interest (ROI) encoder), the all-to-all fusion of four backbone depths in the MSP and the four-depth KPT·Seg FPN, the two-range luminance channels, and the joint-training losses were selected on the basis of preliminary experiments; matched ablations were not performed, so the accuracy–latency trade-off is attributed to the single-pass, shared-backbone design as a whole rather than to these individual choices. Sensor-side evidence: (vii) The hand–eye calibration experiment quantifies the repeatability of two complete procedures, not absolute accuracy against an independent reference (a systematic bias such as the center offset of the perspective image of a sphere, Section 3.3, would not be detected); the rotational repeatability (0.022°) and the working-point repeatability that combines both components (0.30–0.43 mm at 1000 mm, Section 4.1) are likewise measures of repeatability, so the 3D working-point accuracy cannot be stated from this experiment. (viii) The benefit of eliminating the RGB-D registration error is a design rationale and was not quantitatively evaluated against an RGB-D configuration. (ix) Removing the RGB-D registration error does not remove ToF-specific depth artifacts [8,49] (multipath and amplitude-dependent bias on wet, specular tissue, flying pixels at boundaries, invalid depth at saturated pixels, and motion artifacts during the operation); the frequency of invalid depth at the labeled working points was not quantified. The error of the final 3D working point on tissue, which combines depth, calibration, and coordinate transformation, was not measured; establishing it requires independently surveyed targets across the 600–1100 mm range and a tool–target positioning test. Likewise, the end-to-end latency from image acquisition to robot coordinates was not measured; the reported values are network-stage latencies.

## 6. Conclusions

This paper showed that a single-ToF camera, calibrated to the robot with a retroreflective spherical target and read by one shared-backbone network, can estimate the four working points of an evisceration robot in image coordinates on carcasses not used for training, recorded at the same facility with the same camera settings, at a working-point pixel error comparable to that of the two-model pipeline it replaces and with a network-stage latency that stays nearly constant as the number of objects increases (TensorRT FP16).

First, a single-ToF camera was adopted to avoid, by construction, the registration error problem of RGB-D cameras. Judging that a printed checkerboard-type grid is not suitable for reliable feature localization in the low-resolution active-infrared luminance image of the ToF camera, we proposed a calibration target using retroreflective spherical markers and a corresponding calibration procedure. Comparative experiments repeated five times for each condition by changing only the target, its detector, and the exposure setting on the same robot and camera showed that the translational and rotational repeatabilities of the procedure built on the spherical marker were 3.1 and 3.3 times better, respectively, than those of the procedure built on the Radon board (0.374 vs. 1.157 mm and 0.022° vs. 0.073° at *n* = 150), supporting the use of the complete calibration procedure built on the proposed target in this environment.

Second, the integrated multi-task network proposed as a recognition model shares a single DINOv2-S backbone and combines two feature paths, the MSP for detection and the KPT·Seg FPN for keypoints, with a keypoint head initialized from the detection outputs, to simultaneously detect the four working-point classes and estimate the class-specific working points with a single inference of a 43.9 M-parameter network. On five carcasses not used for training, its mean working-point error was 1.83 px compared with 2.01 px for the Serial Network (paired differences of −0.08 to −0.31 px over three training runs; about 2 mm of nominal lateral displacement per pixel at the 1000 mm working distance), and its pooled detection and keypoint AP were within 0.4 AP of those of the Serial Network, while the per-carcass keypoint-AP difference ranged from −0.95 to +2.55 (Table A1); by removing the second model and the crop-and-resize stage, it made the network latency nearly independent of the number of objects. With TensorRT FP16, the batched Serial Network reached 9.44 ms at sixteen objects, and the single-stage YOLOv8l-pose had similar AP and network-stage latency (2.23 ms) but a larger mean working-point error that varied more across training runs (2.45 ± 0.69 px in PyTorch FP32, mainly because of one of the three runs; its median error was similar, 1.03 vs. 1.06 px for the Proposed Network), so the latency advantage holds relative to the two-stage pipeline.

Third, through TensorRT FP16 deployment, a network-stage latency of 2.34 ms (excluding the resize and normalization to 704 × 704, a further 3.80 ms) was achieved with an accuracy loss of about 0.1–0.2 AP and an unchanged mean working-point error (1.83 px); the network stage is therefore within the frame interval of the camera. At the operating threshold, counting every prediction above it (PyTorch FP32), 3.15% of the working points were missed and 4.60% were detected but localized more than 3.90 px from the label, rates similar to those of the Serial Network (2.87% and 4.58%). The 3D positioning accuracy at the tool and closed-loop operation on the line remain to be demonstrated.

## Figures and Tables

**Figure 1:**
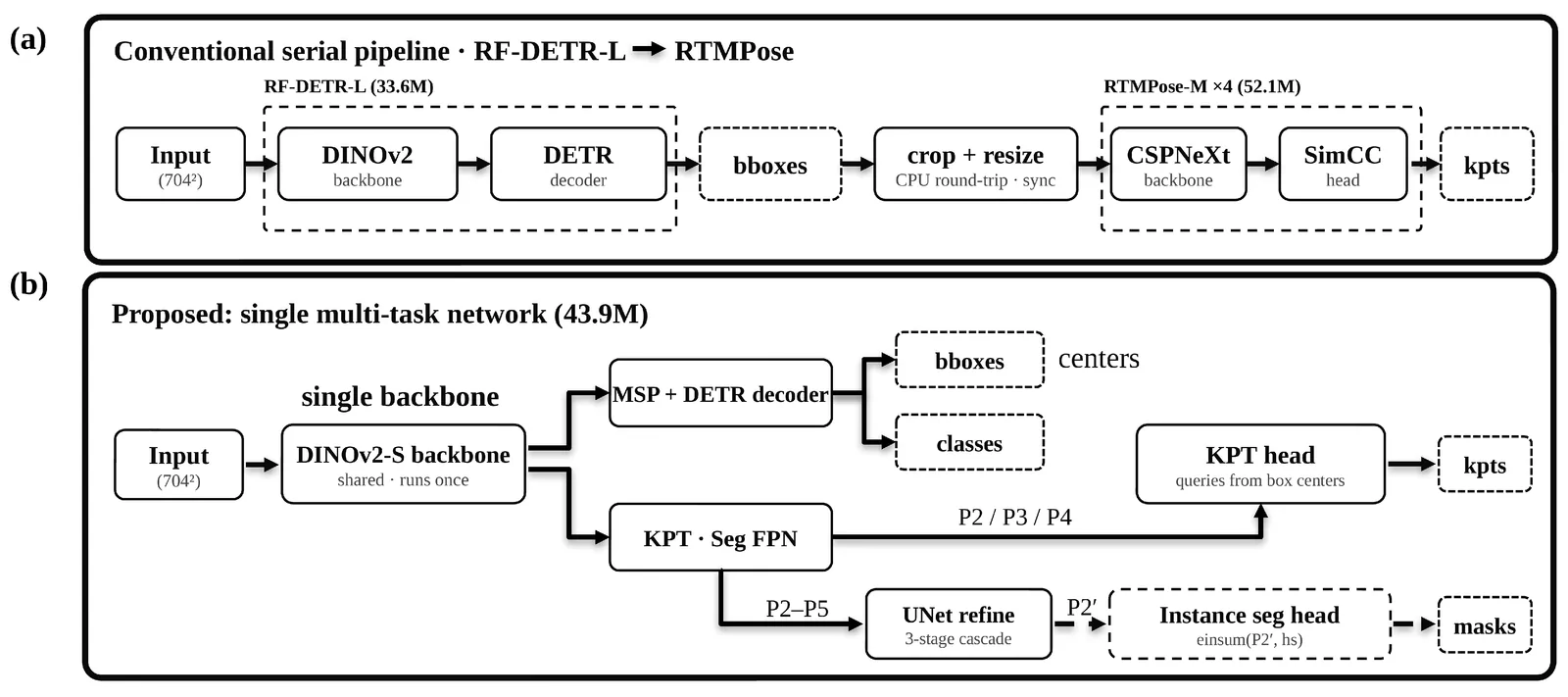
Comparison of the structures of (**a**) the conventional serial configuration of a detector and a pose estimator (hereafter, the Serial Network) and (**b**) the proposed single network (hereafter, the Proposed Network). The instance segmentation head in (**b**), drawn with dashed arrows, is disabled in this work.

**Figure 2:**
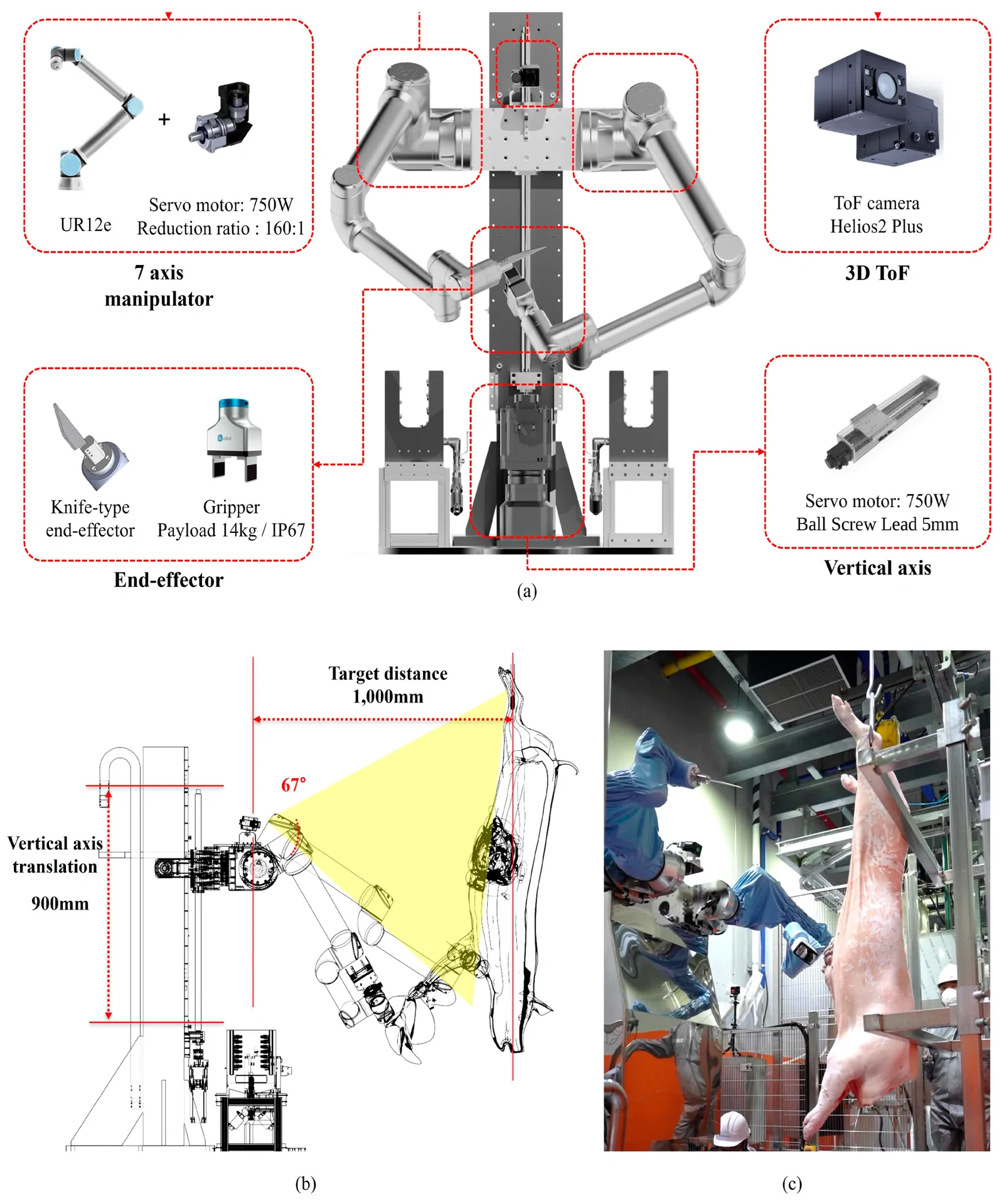
Configuration of the robot system. (**a**) System components: two 7-axis manipulators (UR12e with an additional servo axis), a knife-type end-effector and gripper, a ToF camera (Helios2+), and a vertical axis; (**b**) side view showing the 67° field of view, 1000 mm target working distance, and 900 mm vertical translation; (**c**) system installed on the slaughter line.

**Figure 3:**
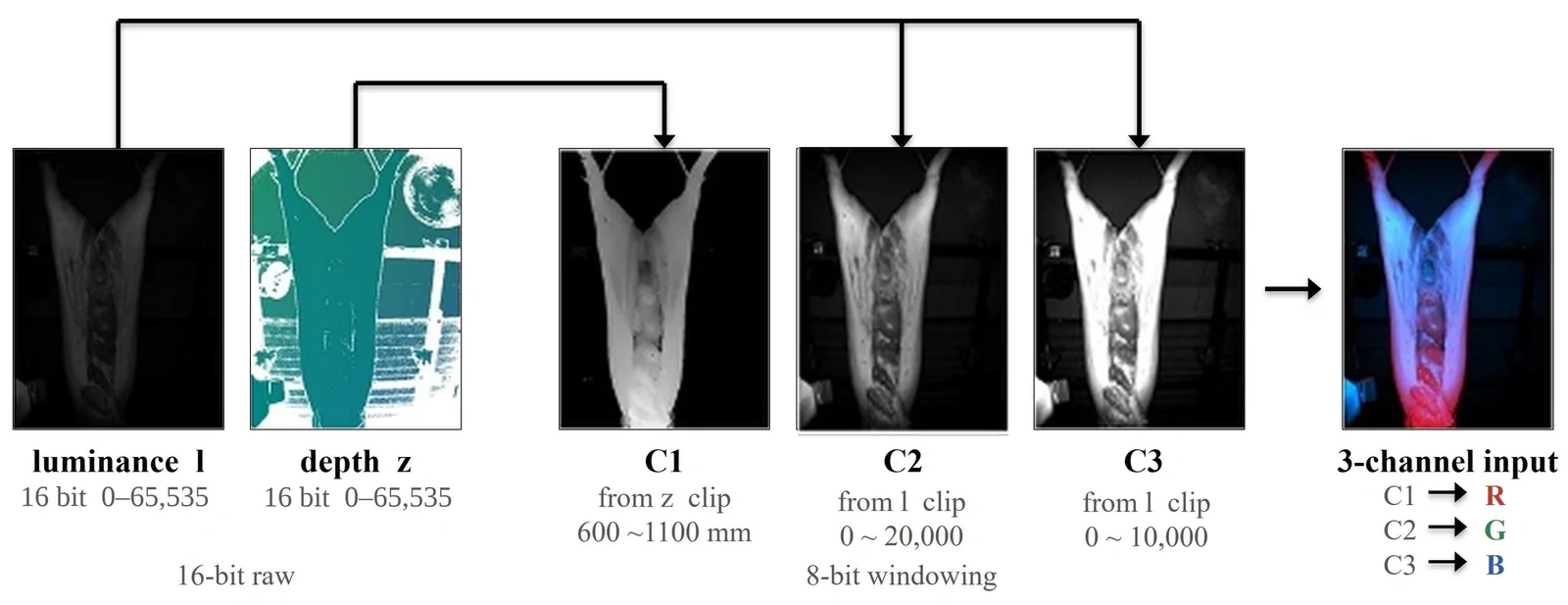
Process of constructing a 3-channel input image from 16-bit raw data of a ToF camera.

**Figure 4:**
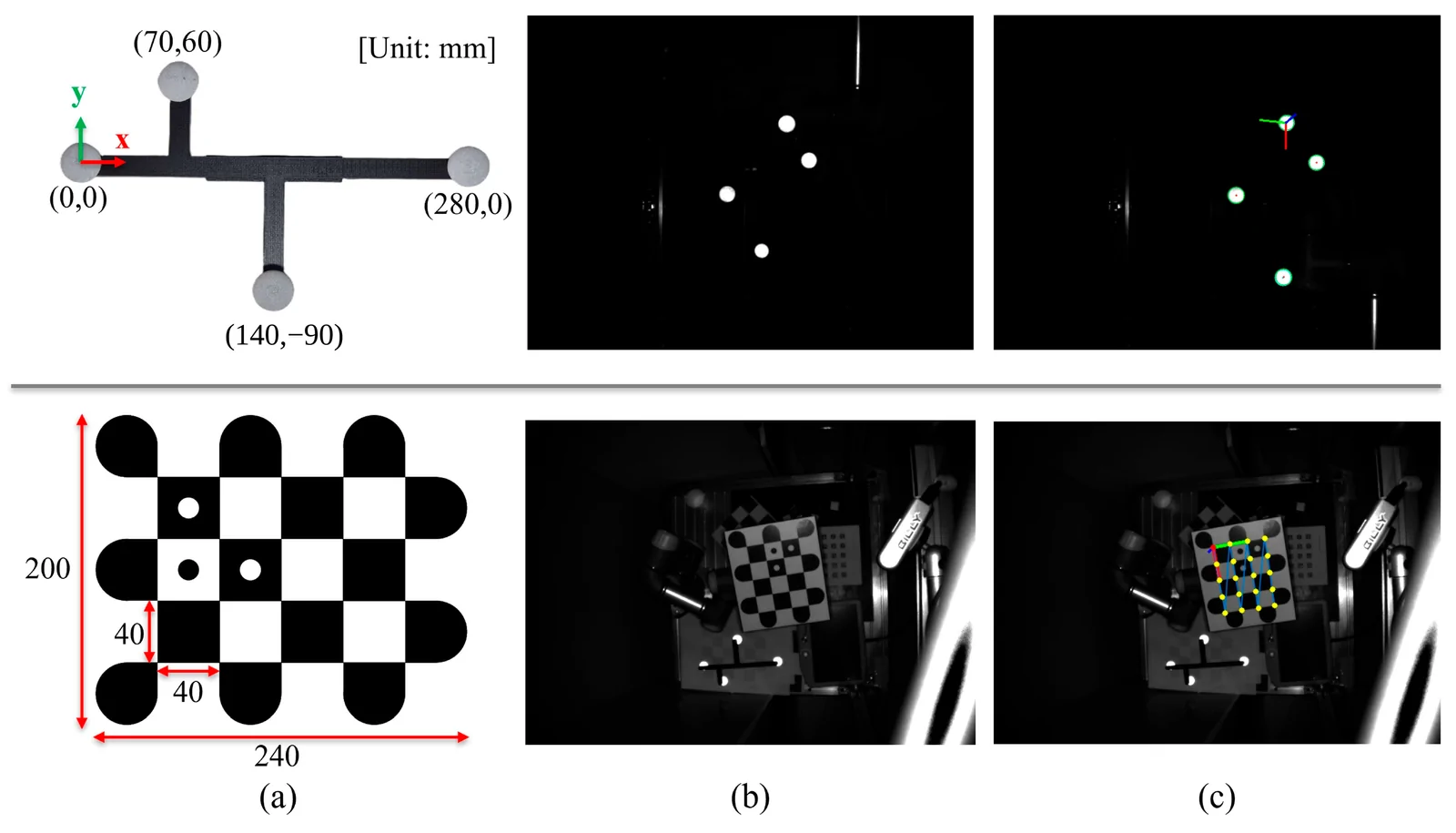
Two calibration targets (top: retroreflective spherical marker, bottom: Radon board). (**a**) Design drawing (unit: mm), (**b**) acquired luminance image, (**c**) detection result.

**Figure 5:**
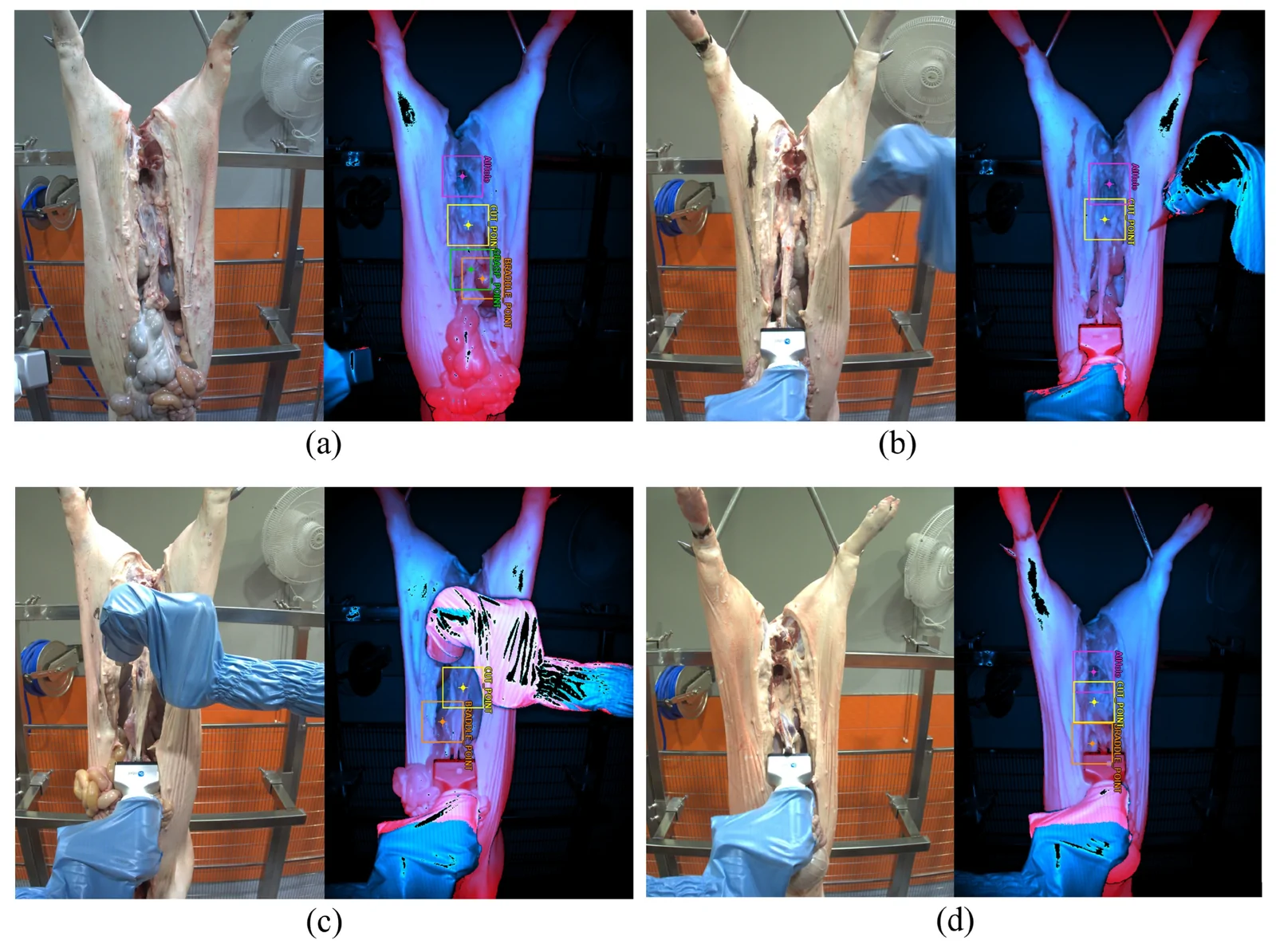
Examples from the real carcass dataset acquired at the testbed. Four frames (**a**–**d**) captured at different time points during the evisceration process are shown; each panel contains an RGB photograph (left) and a 3-channel ToF input image with annotations shown as class-specific colored bounding boxes (right). The label text overlaid in the right panels is the raw class name used in the annotation tool; the colors correspond to BUNG POINT (magenta), CUT POINT (yellow), GRASP POINT (green), and BLADDER POINT (orange).

**Figure 6:**
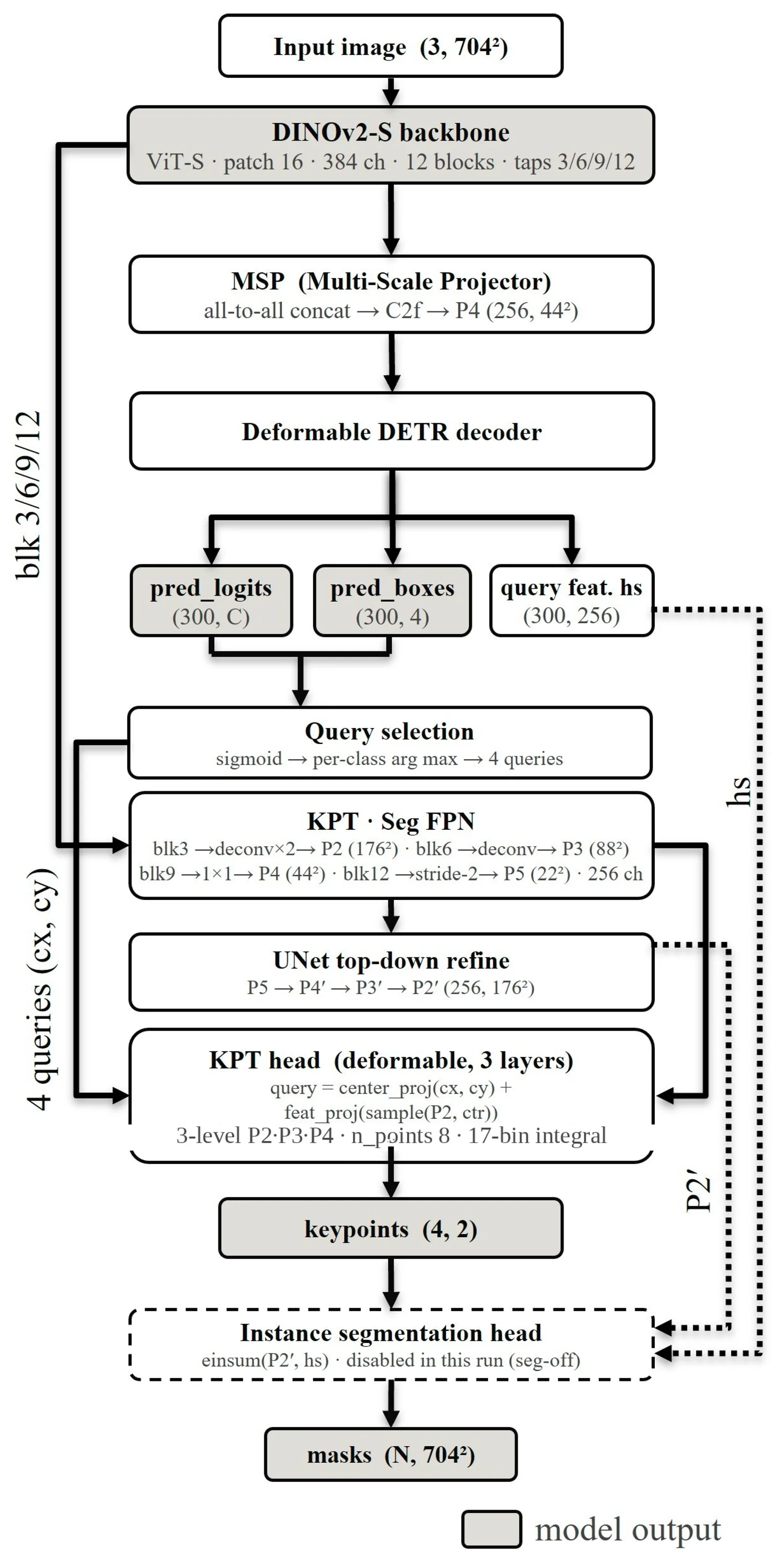
Overall configuration of the Proposed Network. The instance segmentation head is disabled in this training and is indicated by a dashed line.

**Figure 7:**
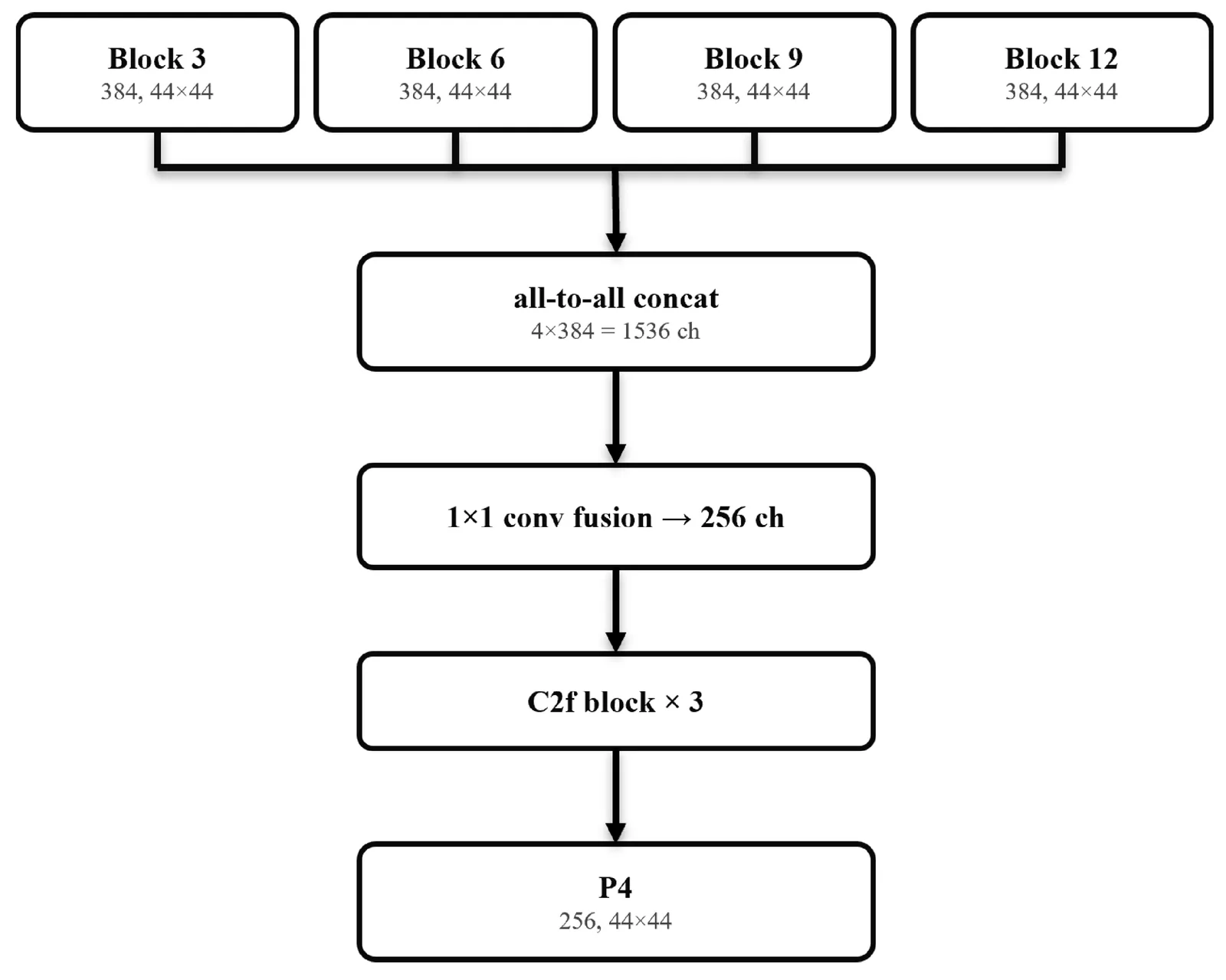
Structure of the Multi-Scale Projector (MSP).

**Figure 8:**
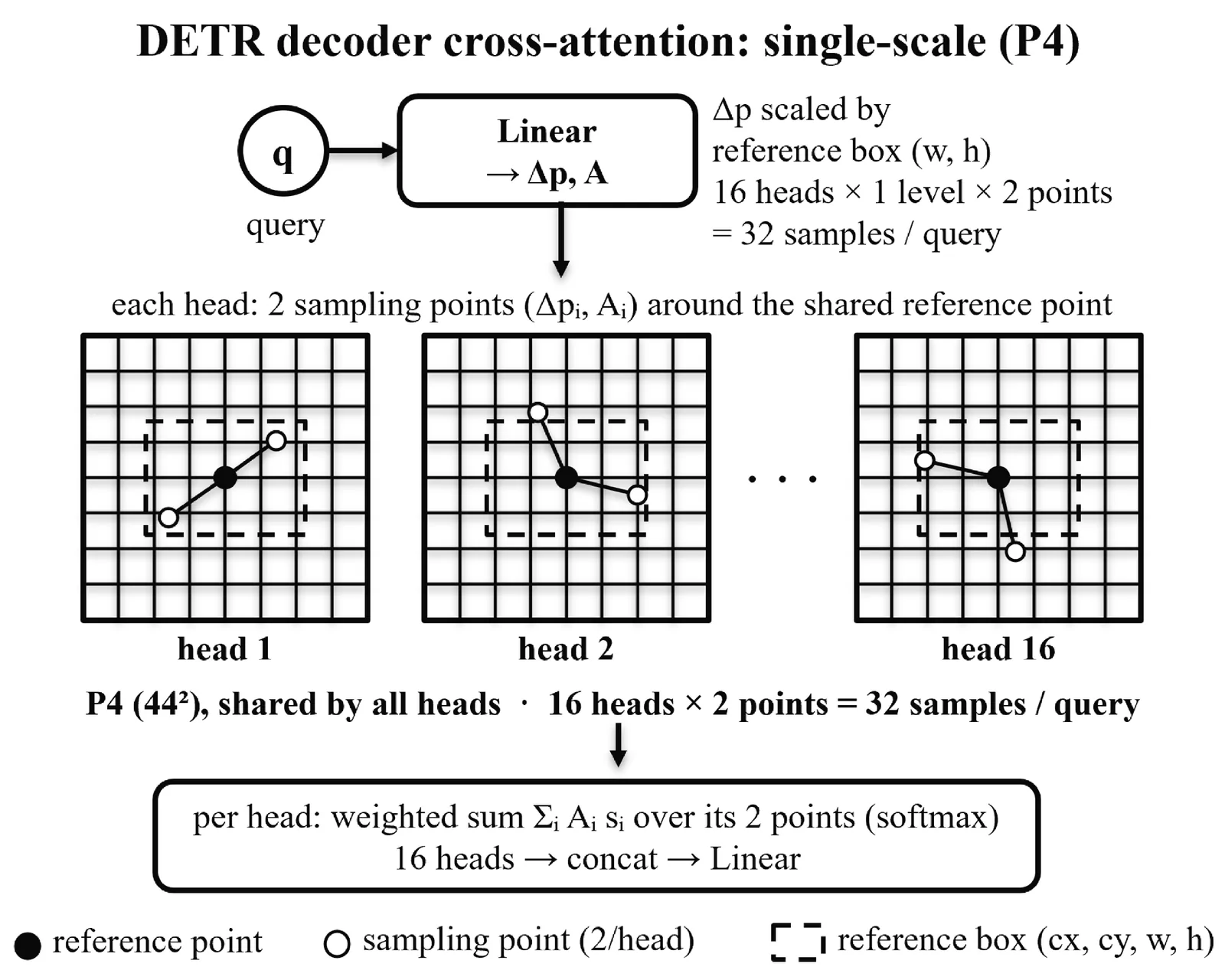
Deformable cross-attention of the DETR decoder on the single-scale P4 feature map.

**Figure 9:**
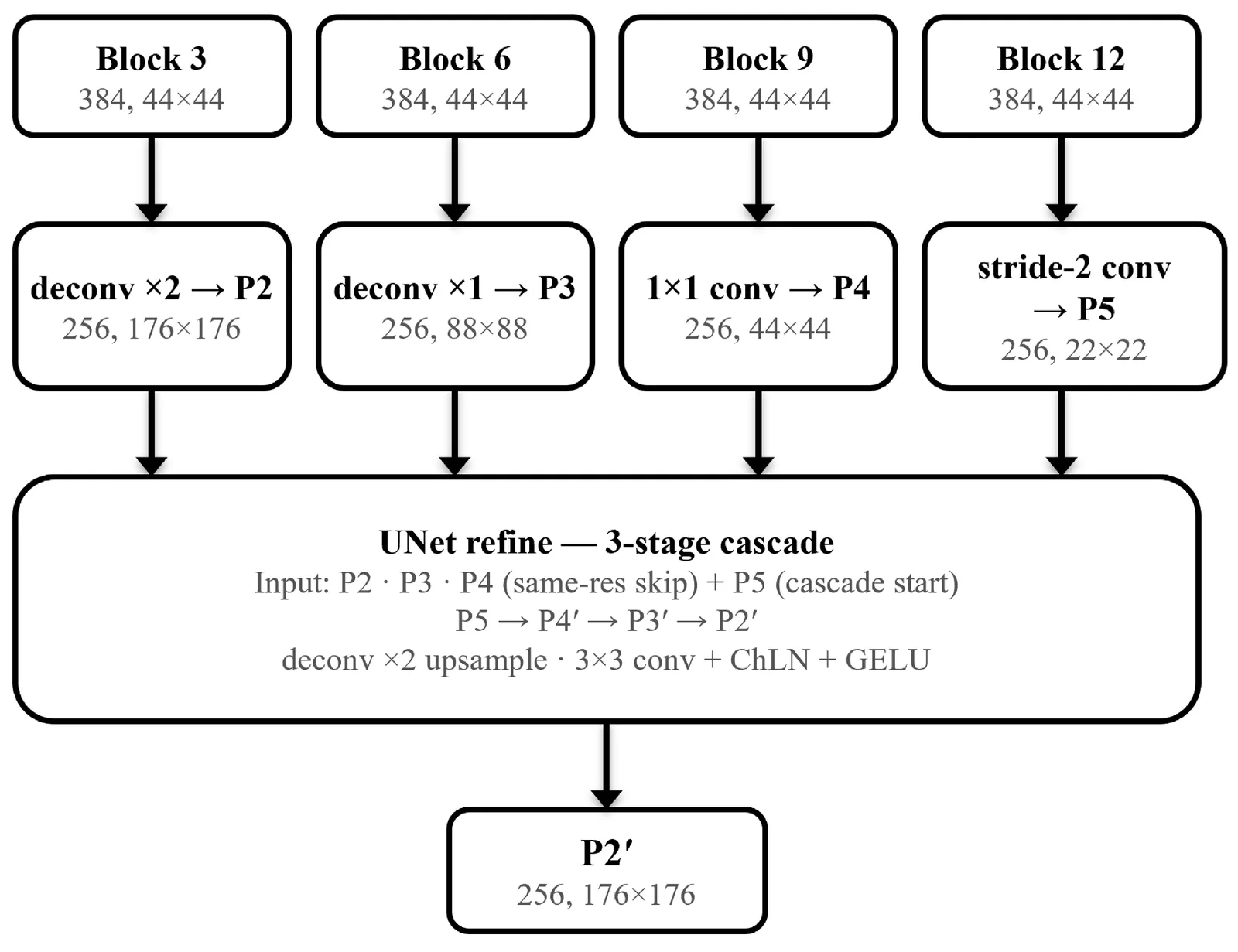
Structure of KPT·Seg FPN and UNet refinement.

**Figure 10:**
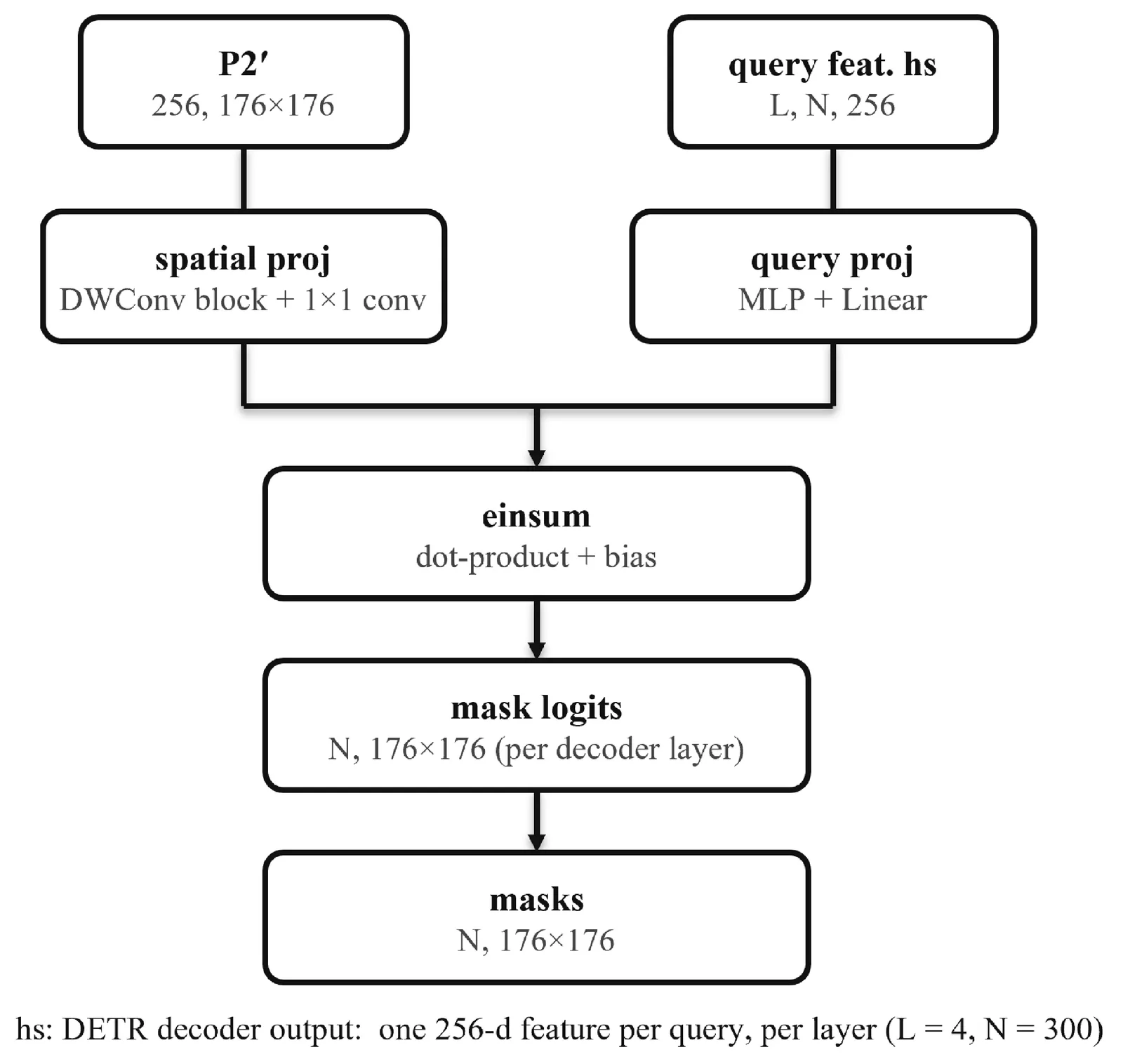
Dot product–based instance segmentation head (selective activation).

**Figure 11:**
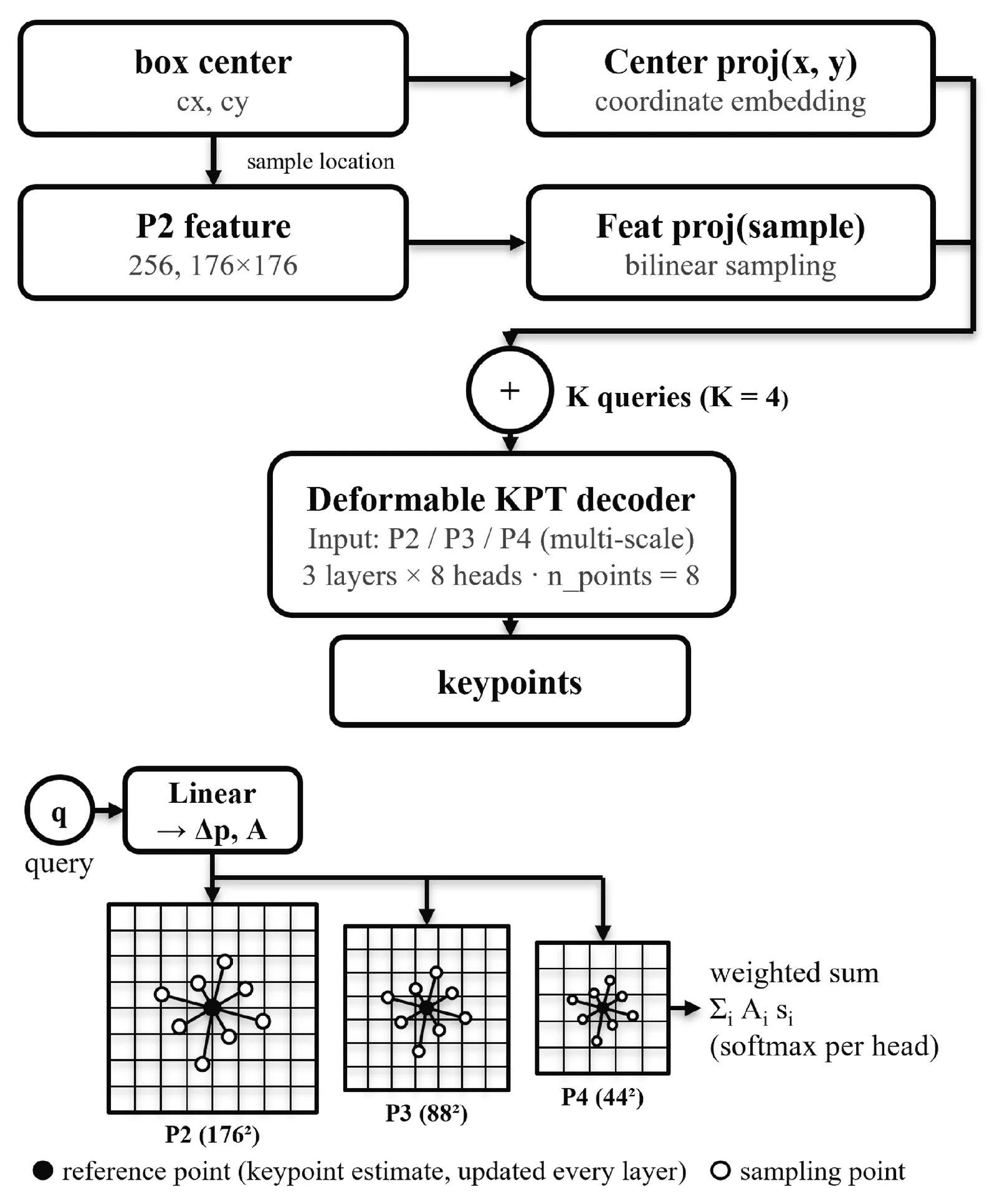
Structure of the keypoint head.

**Figure 12:**
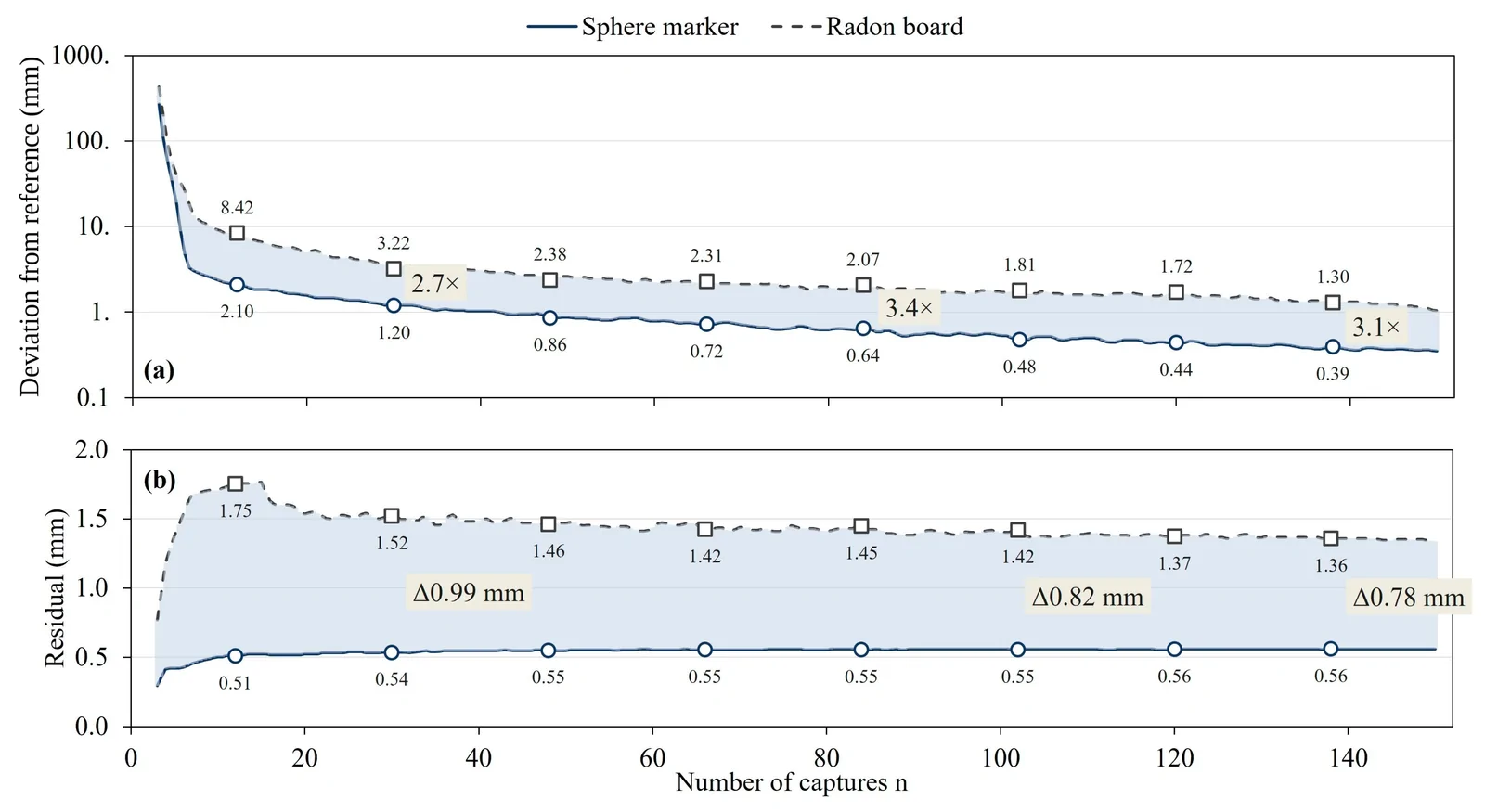
Convergence of the hand–eye solutions according to the number of poses *n* for the spherical marker and the Radon board. (**a**) Distance (mm) between the camera position solved with *n* poses and the reference position; (**b**) residual (mm) of the same solutions. The shading indicates the difference between the two targets. Circles (solid line) and squares (dashed line) denote the spherical marker and the Radon board, respectively.

**Figure 13:**
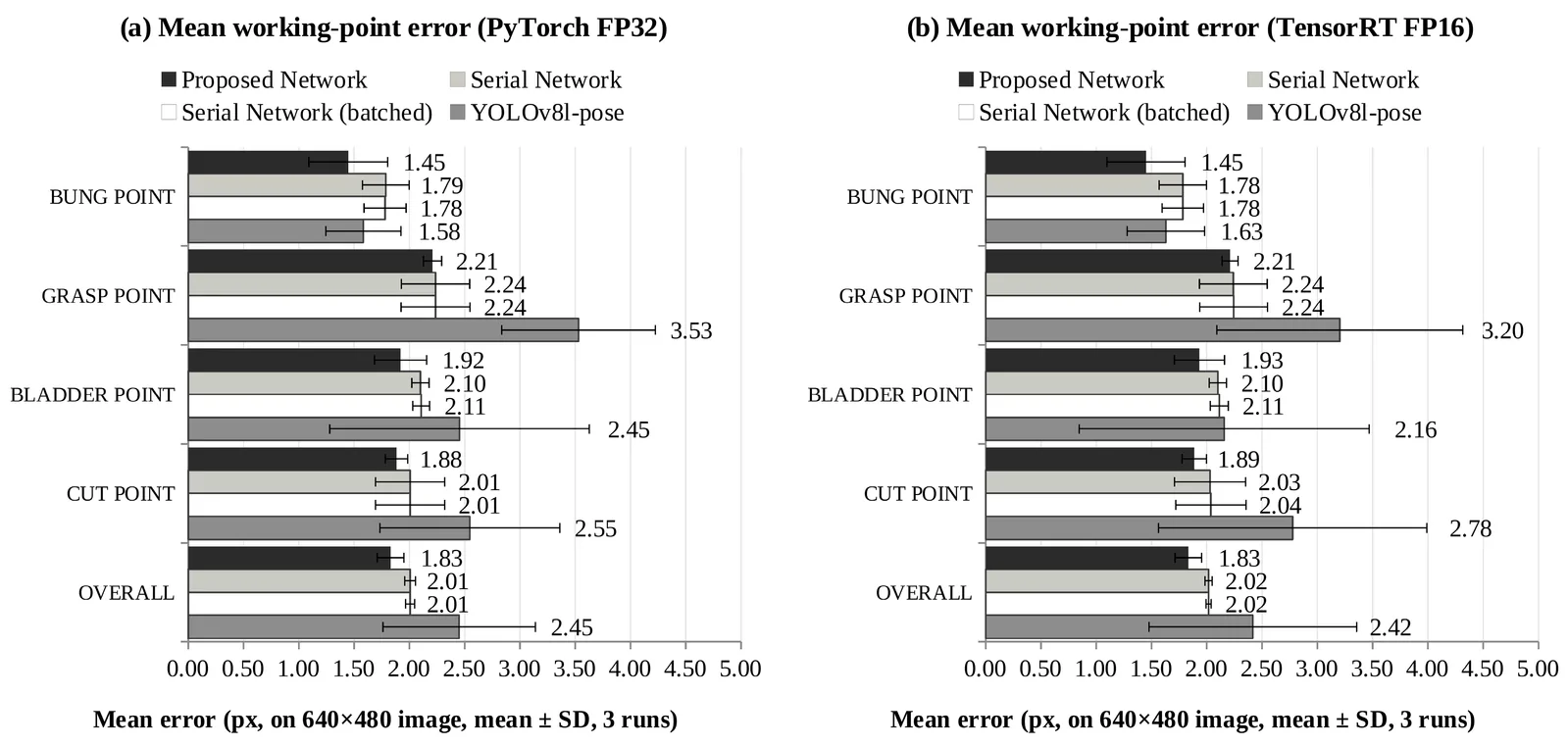
Mean working-point error by class on the test set of five carcasses not used for training (640 × 480 image; mean ± SD over three training runs): (**a**) PyTorch FP32 models; (**b**) TensorRT FP16 engines.

**Figure 14:**
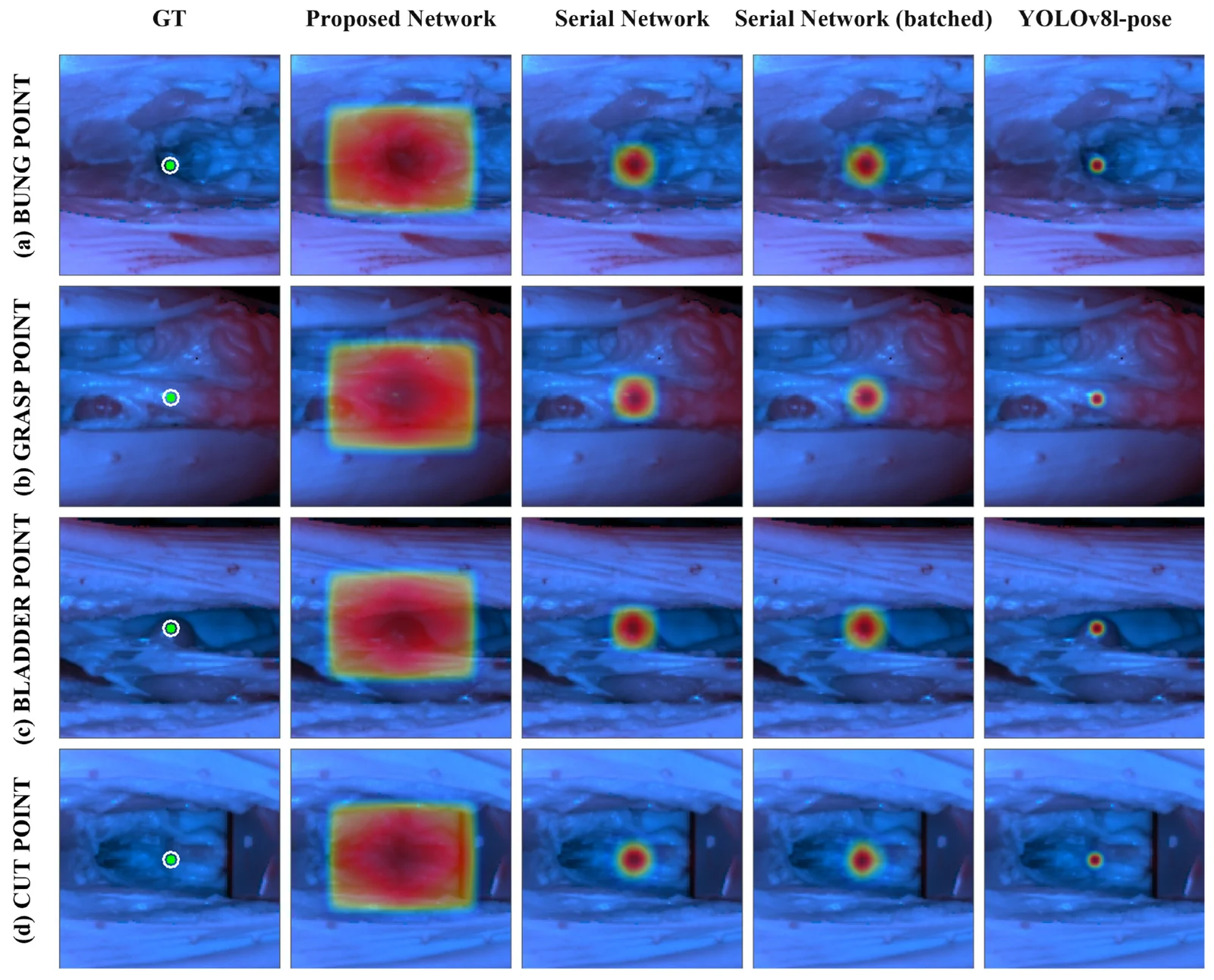
Comparison of the keypoint output distributions for the four working-point classes: (**a**) BUNG POINT, (**b**) GRASP POINT, (**c**) BLADDER POINT, and (**d**) CUT POINT (test set, seed 42). Columns show the ground truth (GT), the Proposed Network (17-bin integral), the Serial Network, the batched Serial Network (SimCC), and YOLOv8l-pose (score-weighted density of the class keypoints of all anchors before non-maximum suppression). The window of the Proposed Network is ±0.07 in normalized coordinates around the distribution center. The overlaid heat map shows the output density (warmer colors indicate higher density), and the green dot marks the ground-truth working point.

**Figure 15:**
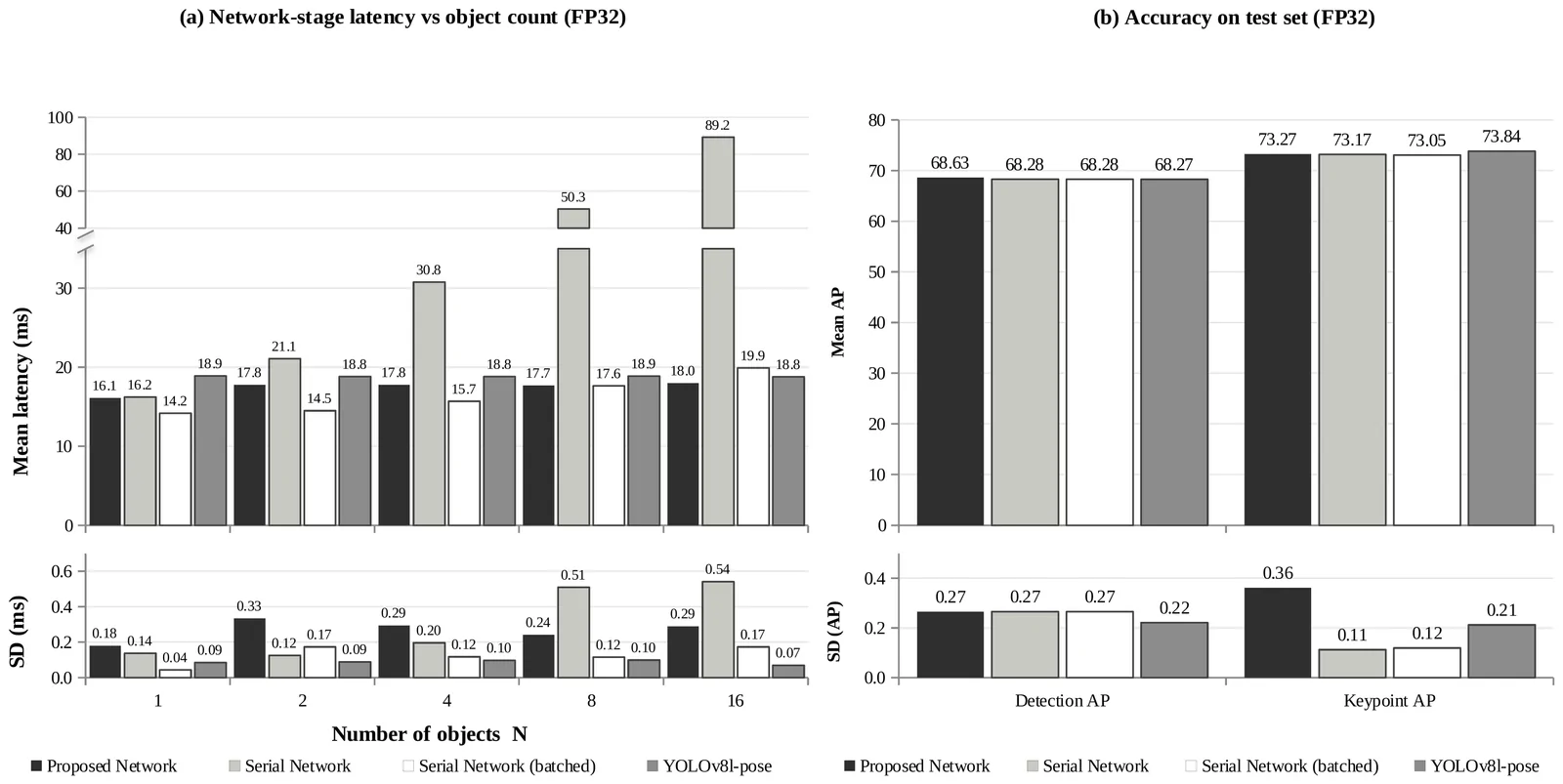
(**a**) Network-stage latency according to the number of objects per frame N and (**b**) detection and keypoint AP on the test set, PyTorch FP32. In each panel, the upper plot shows the mean and the lower plot the SD over three training runs; the latency axis in (**a**) is broken.

**Figure 16:**
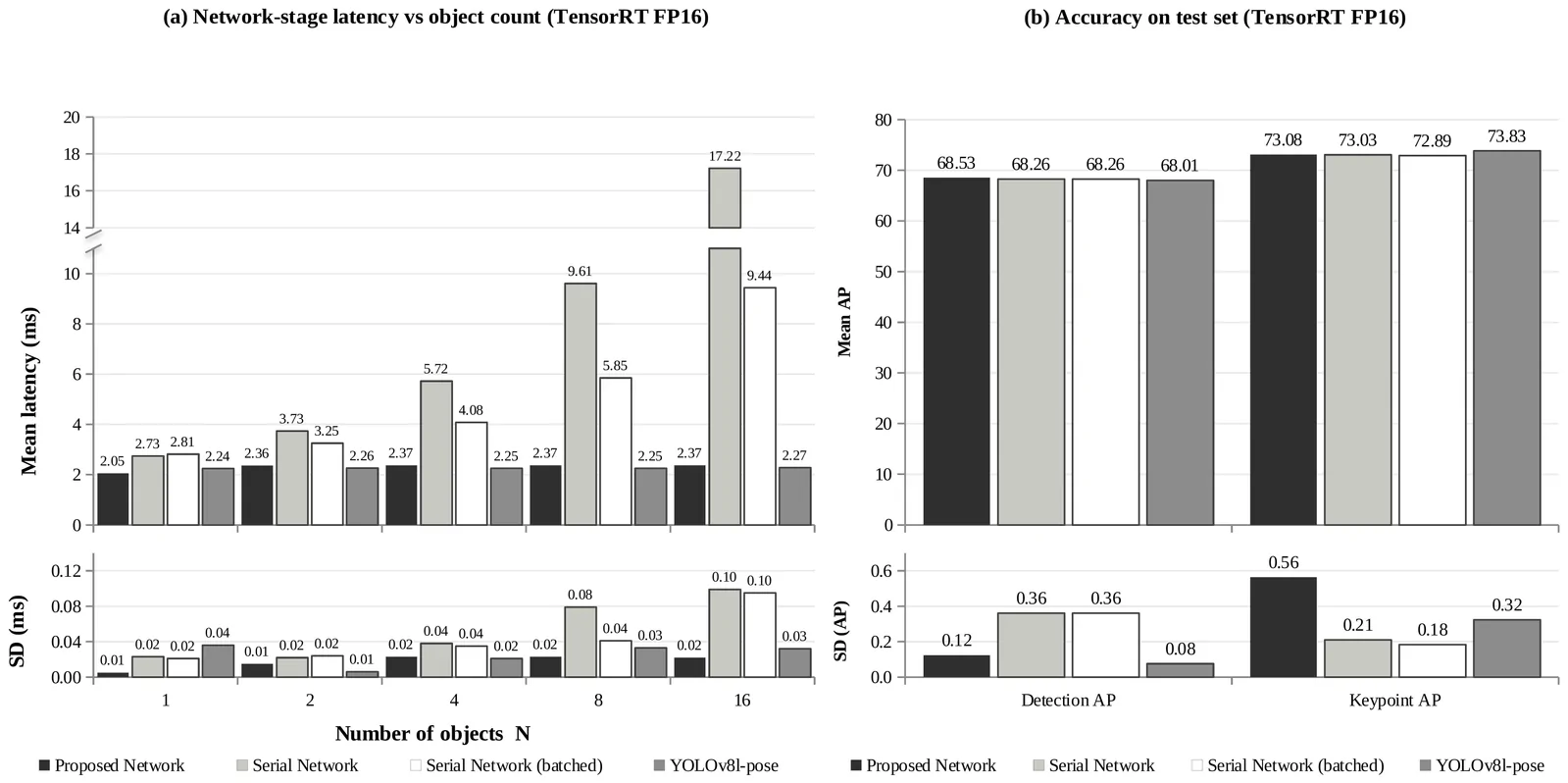
(**a**) Network-stage latency according to the number of objects per frame N and (**b**) detection and keypoint AP on the test set, TensorRT FP16 engines. In each panel, the upper plot shows the mean and the lower plot shows the SD over three training runs; the latency axis in (**a**) is broken.

**Table 1:** Key specifications of the Time-of-Flight (ToF) camera used (LUCID Helios2+).

Item	Specification
Resolution	640 × 480 px
Illumination	Four 850 nm vertical-cavity surface-emitting lasers (VCSELs)
Field of view	67° × 51°
Measurement range	0.3–8.33 m
Frame rate	30 fps (Normal)/10 fps (high dynamic range, HDR)
Exposure time	62.5/250/1000 µs
Output data	x, y, z, luminance (16-bit each)

**Table 2:** Carcasses and frames per partition. The nine original carcasses are used only for training and validation; the test set consists of 100 frames from each of five additional carcasses.

Carcasses	Training Frames	Validation Frames	Test Frames
Nine original carcasses	2677	573	0
p1	0	0	100
p2	0	0	100
p3	0	0	100
p4	0	0	100
p5	0	0	100
Total	2677	573	500

**Table 3:** COCO summary metrics on the test set of five carcasses not used for training (500 frames, PyTorch FP32, mean ± SD over three training runs). Difference = Proposed − Serial.

Metric	Proposed Network	Serial Network	Serial Network (Batched)	YOLOv8l-Pose	Difference (Proposed−Serial)
Detection AP	68.63 ± 0.27	68.28 ± 0.27	68.28 ± 0.27	68.27 ± 0.22	+0.35
Detection AP50	96.43 ± 0.13	96.45 ± 0.15	96.45 ± 0.15	95.71 ± 0.07	−0.02
Detection AP75	75.54 ± 1.27	75.29 ± 0.29	75.29 ± 0.29	76.34 ± 0.26	+0.25
Keypoint AP	73.27 ± 0.36	73.17 ± 0.11	73.05 ± 0.12	73.84 ± 0.21	+0.10
Keypoint AP50	90.26 ± 0.60	90.80 ± 0.26	90.64 ± 0.36	90.61 ± 0.54	−0.54
Keypoint AP75	76.78 ± 0.23	75.98 ± 0.16	75.72 ± 0.37	77.80 ± 0.47	+0.80

**Table 4:** Mean working-point error by class (px, PyTorch FP32, mean ± SD over three training runs, all ground-truth points included).

Class	Proposed Network	Serial Network	Serial Network (Batched)	YOLOv8l-Pose	Difference (Proposed−Serial)
BUNG POINT	1.45 ± 0.36	1.79 ± 0.21	1.78 ± 0.19	1.58 ± 0.34	−0.34
GRASP POINT	2.21 ± 0.08	2.24 ± 0.31	2.24 ± 0.31	3.53 ± 0.69	−0.03
BLADDER POINT	1.92 ± 0.24	2.10 ± 0.08	2.11 ± 0.08	2.45 ± 1.17	−0.18
CUT POINT	1.88 ± 0.10	2.01 ± 0.31	2.01 ± 0.31	2.55 ± 0.81	−0.13
Overall (all instances)	1.83 ± 0.12	2.01 ± 0.05	2.01 ± 0.04	2.45 ± 0.69	−0.18

**Table 5:** Paired comparison of the working-point error with the Serial Network for each training seed (carcass-level bootstrap with 2000 resamples, 90% confidence interval; differences are computed from unrounded values).

Network	Seed	Mean Error (px)	Difference (px)	90% CI (px)
Proposed Network	42	1.96	−0.08	[−0.32, +0.16]
Proposed Network	43	1.72	−0.31	[−0.59, −0.07]
Proposed Network	44	1.81	−0.14	[−0.36, +0.05]
Serial Network	42	2.04	Reference	—
Serial Network	43	2.03	Reference	—
Serial Network	44	1.95	Reference	—
Serial Network (batched)	42	2.03	−0.01	[−0.02, 0.00]
Serial Network (batched)	43	2.03	+0.01	[0.00, +0.01]
Serial Network (batched)	44	1.96	+0.01	[0.00, +0.02]
YOLOv8l-pose	42	2.14	+0.10	[−0.36, +0.57]
YOLOv8l-pose	43	3.24	+1.21	[+0.37, +2.12]
YOLOv8l-pose	44	1.97	+0.02	[−0.25, +0.35]

**Table 6:** Network-stage latency on an RTX 5090 (ms, mean ± SD over three training runs). Deployed path = the four class-wise queries for the Proposed Network and the objects detected in the test frames for the other networks (Section 3.11.2); N = number of objects per frame forced.

Objects per Frame	Proposed Network	Serial Network	Serial Network (Batched)	YOLOv8l-Pose	Proposed/Serial	Proposed/Serial (Batched)
TensorRT FP16						
Deployed path	2.34 ± 0.01	4.11 ± 0.04	3.40 ± 0.02	2.23 ± 0.05	0.57	0.69
N = 1	2.05 ± 0.01	2.73 ± 0.02	2.81 ± 0.02	2.24 ± 0.04	0.75	0.73
N = 2	2.36 ± 0.01	3.73 ± 0.02	3.25 ± 0.02	2.26 ± 0.01	0.63	0.73
N = 4	2.37 ± 0.02	5.72 ± 0.04	4.08 ± 0.04	2.25 ± 0.02	0.41	0.58
N = 8	2.37 ± 0.02	9.61 ± 0.08	5.85 ± 0.04	2.25 ± 0.03	0.25	0.41
N = 16	2.37 ± 0.02	17.22 ± 0.10	9.44 ± 0.10	2.27 ± 0.03	0.14	0.25
PyTorch FP32						
Deployed path	17.87 ± 0.41	22.92 ± 0.53	14.74 ± 0.24	18.88 ± 0.19	0.78	1.21
N = 1	16.11 ± 0.18	16.22 ± 0.14	14.17 ± 0.04	18.91 ± 0.09	0.99	1.14
N = 2	17.77 ± 0.33	21.06 ± 0.12	14.48 ± 0.17	18.81 ± 0.09	0.84	1.23
N = 4	17.77 ± 0.29	30.76 ± 0.20	15.68 ± 0.12	18.81 ± 0.10	0.58	1.13
N = 8	17.69 ± 0.24	50.31 ± 0.51	17.64 ± 0.12	18.88 ± 0.10	0.35	1.00
N = 16	17.99 ± 0.29	89.19 ± 0.54	19.90 ± 0.17	18.78 ± 0.07	0.20	0.90

**Table 7:** Accuracy of the PyTorch FP32 models and of the TensorRT FP16 engines used for the latency measurements (PyTorch FP32/TensorRT FP16, change in parentheses, mean over three training runs; changes are computed from unrounded values).

Network	Detection AP	Keypoint AP	Mean Error (px)	Median Error (px)
Proposed Network	68.63/68.53 (−0.10)	73.27/73.08 (−0.20)	1.83/1.83 (0.00)	1.06/1.07 (+0.01)
Serial Network	68.28/68.26 (−0.02)	73.17/73.03 (−0.14)	2.01/2.02 (+0.01)	1.10/1.10 (0.00)
Serial Network (batched)	68.28/68.26 (−0.02)	73.05/72.89 (−0.17)	2.01/2.02 (+0.01)	1.10/1.10 (0.00)
YOLOv8l-pose	68.27/68.01 (−0.26)	73.84/73.83 (−0.02)	2.45/2.42 (−0.03)	1.03/1.04 (+0.01)

**Table 8:** Working-point outcomes at the operating threshold of 0.3, counting every prediction above the threshold (Section 3.11.2) (%, PyTorch FP32, mean ± SD over three training runs; the false-working-point rate equals 100−precision).

Class	Network	Precision	Recall	Miss Rate	False-Working-Point Rate
BUNG POINT	Proposed Network	95.91 ± 0.16	95.15 ± 0.40	1.41 ± 0.16	4.09 ± 0.16
	Serial Network	95.73 ± 0.28	94.97 ± 0.46	1.24 ± 0.31	4.27 ± 0.28
	Serial Network (batched)	95.55 ± 0.41	94.80 ± 0.41	1.24 ± 0.31	4.45 ± 0.41
	YOLOv8l-pose	95.98 ± 0.01	94.80 ± 0.15	1.85 ± 0.27	4.02 ± 0.01
GRASP POINT	Proposed Network	87.93 ± 0.19	86.90 ± 0.23	4.93 ± 0.45	12.07 ± 0.19
	Serial Network	88.12 ± 0.77	87.55 ± 0.39	4.54 ± 0.98	11.88 ± 0.77
	Serial Network (batched)	88.12 ± 0.77	87.55 ± 0.39	4.54 ± 0.98	11.88 ± 0.77
	YOLOv8l-pose	89.53 ± 0.74	87.55 ± 0.00	6.49 ± 0.81	10.47 ± 0.74
BLADDER POINT	Proposed Network	96.33 ± 0.40	94.02 ± 0.40	2.79 ± 0.00	3.67 ± 0.40
	Serial Network	96.08 ± 0.82	94.42 ± 0.40	2.92 ± 0.46	3.92 ± 0.82
	Serial Network (batched)	96.08 ± 0.82	94.42 ± 0.40	2.92 ± 0.46	3.92 ± 0.82
	YOLOv8l-pose	96.50 ± 1.17	95.09 ± 1.28	2.12 ± 0.23	3.50 ± 1.17
CUT POINT	Proposed Network	93.85 ± 1.03	91.89 ± 0.90	3.79 ± 0.23	6.15 ± 1.03
	Serial Network	94.30 ± 0.92	92.34 ± 1.31	3.27 ± 0.68	5.70 ± 0.92
	Serial Network (batched)	94.30 ± 0.92	92.34 ± 1.31	3.27 ± 0.68	5.70 ± 0.92
	YOLOv8l-pose	94.60 ± 0.48	92.56 ± 0.56	3.20 ± 0.26	5.40 ± 0.48
All classes	Proposed Network	93.75 ± 0.38	92.25 ± 0.30	3.15 ± 0.07	6.25 ± 0.38
	Serial Network	93.84 ± 0.27	92.55 ± 0.50	2.87 ± 0.41	6.16 ± 0.27
	Serial Network (batched)	93.79 ± 0.18	92.50 ± 0.46	2.87 ± 0.41	6.21 ± 0.18
	YOLOv8l-pose	94.38 ± 0.28	92.70 ± 0.11	3.25 ± 0.16	5.62 ± 0.28

**Table 9:** Working-point error distribution by class (px, PyTorch FP32, mean ± SD over three training runs, all ground-truth points included).

Class	Network	Median	95th Percentile	99th Percentile
BUNG POINT	Proposed Network	0.66 ± 0.07	3.68 ± 0.05	6.45 ± 0.51
	Serial Network	0.64 ± 0.04	3.82 ± 0.06	7.01 ± 0.32
	Serial Network (batched)	0.64 ± 0.04	3.86 ± 0.07	7.04 ± 0.27
	YOLOv8l-pose	0.71 ± 0.05	3.76 ± 0.06	6.76 ± 0.53
GRASP POINT	Proposed Network	1.37 ± 0.03	6.13 ± 0.18	16.65 ± 1.01
	Serial Network	1.37 ± 0.10	5.36 ± 0.45	14.92 ± 2.22
	Serial Network (batched)	1.37 ± 0.11	5.31 ± 0.42	14.92 ± 2.22
	YOLOv8l-pose	1.25 ± 0.02	7.06 ± 0.21	33.88 ± 24.23
BLADDER POINT	Proposed Network	1.00 ± 0.03	3.81 ± 0.33	17.77 ± 7.91
	Serial Network	1.05 ± 0.08	3.90 ± 0.38	28.41 ± 8.87
	Serial Network (batched)	1.07 ± 0.08	4.04 ± 0.25	28.41 ± 8.87
	YOLOv8l-pose	1.02 ± 0.02	3.81 ± 0.31	8.57 ± 3.31
CUT POINT	Proposed Network	1.25 ± 0.06	4.46 ± 0.21	10.08 ± 0.60
	Serial Network	1.29 ± 0.03	4.32 ± 0.06	9.47 ± 1.81
	Serial Network (batched)	1.30 ± 0.03	4.32 ± 0.06	9.47 ± 1.81
	YOLOv8l-pose	1.18 ± 0.02	4.19 ± 0.04	9.00 ± 0.90

**Table 10:** AP for different OKS definitions (mean ± SD over three training runs). Tolerance = distance at which the OKS equals 0.5.

Condition	Tolerance (px)	Proposed Network	Serial Network	Serial Network (Batched)	YOLOv8l-Pose	Difference (Proposed−Serial)
Keypoint AP, OKS area (σ = 0.079)						
16 × 16	2.98	62.45 ± 0.69	62.27 ± 0.53	62.12 ± 0.53	63.04 ± 0.29	+0.18
22 × 20 (used)	3.90	73.27 ± 0.36	73.17 ± 0.11	73.05 ± 0.12	73.84 ± 0.21	+0.10
32 × 32	5.95	85.98 ± 0.09	86.18 ± 0.20	86.10 ± 0.20	86.14 ± 0.10	−0.20
50 × 50	9.30	93.72 ± 0.03	93.67 ± 0.13	93.66 ± 0.14	93.30 ± 0.17	+0.05
Keypoint AP, σ (area 22 × 20)						
σ = 0.025	1.23	27.07 ± 1.19	27.04 ± 0.79	27.01 ± 0.75	25.66 ± 0.55	+0.03
σ = 0.05	2.47	54.41 ± 0.86	54.35 ± 0.70	54.22 ± 0.71	54.88 ± 0.45	+0.06
σ = 0.079 (used)	3.90	73.27 ± 0.36	73.17 ± 0.11	73.05 ± 0.12	73.84 ± 0.21	+0.10
σ = 0.107	5.29	82.98 ± 0.05	82.95 ± 0.21	82.87 ± 0.27	83.05 ± 0.05	+0.03
Detection AP, box size (ground-truth and predicted boxes)						
16 × 16	—	60.40 ± 0.26	60.12 ± 0.51	60.12 ± 0.51	60.08 ± 0.26	+0.28
22 × 20 (used)	—	68.69 ± 0.29	68.33 ± 0.26	68.33 ± 0.26	68.30 ± 0.27	+0.36
32 × 32	—	79.11 ± 0.19	78.93 ± 0.12	78.93 ± 0.12	78.62 ± 0.03	+0.18
50 × 50	—	87.10 ± 0.11	87.02 ± 0.19	87.02 ± 0.19	86.82 ± 0.06	+0.08

— not applicable (the tolerance is defined only for the OKS-based keypoint AP). The detection AP for 22 × 20 differs slightly from Table 3 because the predicted boxes were also resized.

**Figure A1:**
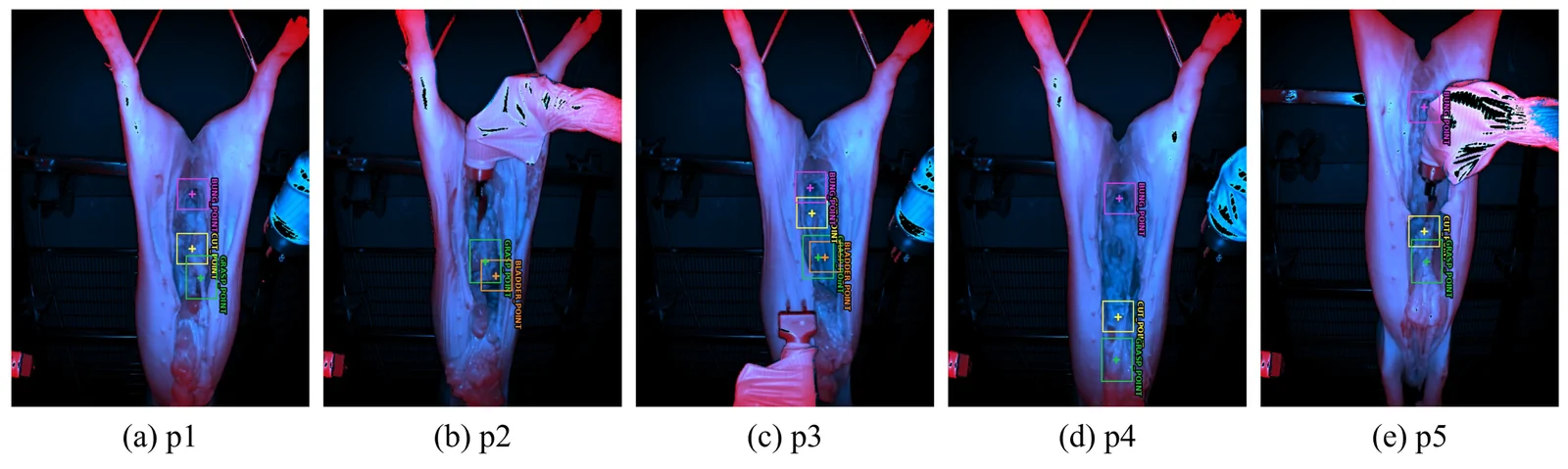
Examples of the test set: one frame from each of the five carcasses (**a**–**e**) not used for training (three-channel ToF input). The boxes are drawn for display around the ground-truth working points; evaluation uses fixed 22 × 20 px boxes. Colors: BUNG POINT (magenta), GRASP POINT (green), BLADDER POINT (orange), and CUT POINT (yellow).

**Figure A2:**
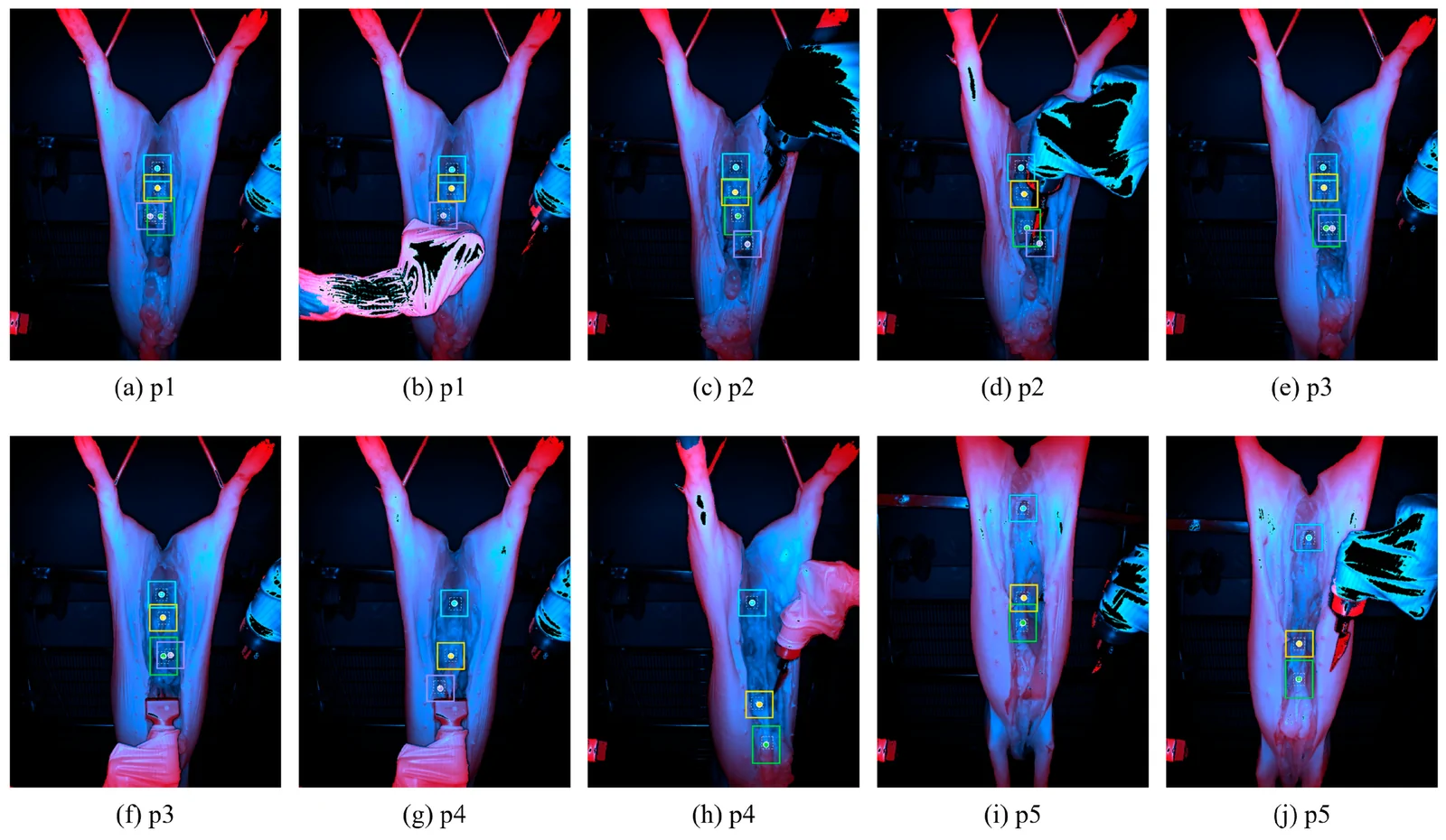
Final output of the Proposed Network (seed 42, PyTorch FP32) on two test frames from each of the five test carcasses (**a**–**j**). Predictions (detection score > 0.3, highest-scoring output per class) are shown as colored points with boxes drawn at a fixed display size; ground truth is shown as gray dashed 22 × 20 px boxes with white crosses. Colors: BUNG POINT (sky blue), GRASP POINT (green), BLADDER POINT (purple), and CUT POINT (yellow).

## Data Availability

The data presented in this study are available from the corresponding author upon reasonable request; the data and the training code are not publicly available due to commercial confidentiality.

## Appendix A. Test-Set Examples and Final Outputs

Figure A1 shows one test frame from each of the five carcasses not used for training. Figure A2 shows the final output of the Proposed Network on two test frames from each of these carcasses. In a single inference pass, the bounding boxes of the working-point classes present in each frame and the corresponding working-point coordinates are produced simultaneously, without intermediate cropping or a second model call.

Because evisceration proceeds in stages, the four working points do not necessarily appear simultaneously in a single frame; classes that are not yet exposed at that stage or that are occluded by the operator or tools have no labels (Section 3.4). The frames shown are the two frames with the most ground-truth working points among ten randomly drawn test frames of each carcass; all 35 ground-truth working points in these frames were output, with a maximum error per frame of 0.47–4.85 px. These frames were selected to illustrate the output rather than to represent the error distribution of the test set; the depth at saturated pixels is invalid, so the 2D result alone does not guarantee a valid 3D working point there (limitation (ix) in Section 5).

## Appendix B. Results per Test Carcass and per Class

Table A1 lists the detection AP, keypoint AP, and mean working-point error of each network for each of the five test carcasses; the last row of each block gives the mean ± SD across carcasses. Table A2 gives the class-wise detection AP and keypoint AP of each network.

**Table A1:** Results for each test carcass (mean ± SD over three training runs; in the rows “Across carcasses”, mean ± SD across the five carcasses).

Carcass	Proposed Network	Serial Network	Serial Network (Batched)	YOLOv8l-Pose	Difference (Proposed−Serial)
Detection AP					
p1	72.06 ± 0.26	71.90 ± 0.47	71.90 ± 0.47	69.88 ± 0.79	+0.16
p2	64.54 ± 1.28	63.55 ± 0.19	63.55 ± 0.19	67.10 ± 0.20	+0.99
p3	71.77 ± 0.09	71.16 ± 1.48	71.16 ± 1.48	70.96 ± 1.08	+0.61
p4	60.32 ± 1.25	59.33 ± 1.14	59.33 ± 1.14	58.74 ± 2.05	+0.99
p5	70.07 ± 0.23	69.98 ± 0.15	69.98 ± 0.15	69.67 ± 0.68	+0.09
Across carcasses	67.75 ± 5.14	67.19 ± 5.50	67.19 ± 5.50	67.27 ± 4.97	+0.56
Keypoint AP					
p1	77.87 ± 1.07	77.42 ± 0.47	77.37 ± 0.50	76.06 ± 0.67	+0.45
p2	67.75 ± 1.04	67.19 ± 0.92	66.92 ± 0.68	72.22 ± 0.61	+0.56
p3	78.19 ± 0.14	77.87 ± 1.74	77.73 ± 1.50	77.82 ± 0.83	+0.32
p4	62.21 ± 1.74	59.66 ± 1.39	59.73 ± 1.31	60.48 ± 1.15	+2.55
p5	73.61 ± 0.57	74.56 ± 0.42	74.60 ± 0.36	74.74 ± 0.67	−0.95
Across carcasses	71.93 ± 6.88	71.34 ± 7.81	71.27 ± 7.78	72.27 ± 6.90	+0.59
Mean working-point error (px)					
p1	2.10 ± 0.02	1.87 ± 0.21	1.88 ± 0.21	2.71 ± 1.26	+0.23
p2	1.72 ± 0.04	1.79 ± 0.05	1.80 ± 0.05	1.62 ± 0.03	−0.07
p3	1.43 ± 0.22	1.54 ± 0.04	1.54 ± 0.05	2.14 ± 0.27	−0.11
p4	2.33 ± 0.59	2.86 ± 0.32	2.84 ± 0.31	4.07 ± 2.01	−0.53
p5	1.64 ± 0.04	2.13 ± 0.09	2.14 ± 0.09	2.03 ± 0.31	−0.49
Across carcasses	1.84 ± 0.36	2.04 ± 0.50	2.04 ± 0.50	2.51 ± 0.95	−0.20

**Table A2:** Class-wise detection AP and keypoint AP on the test set (mean ± SD over three training runs).

Class	Proposed Network	Serial Network	Serial Network (Batched)	YOLOv8l-Pose	Difference (Proposed−Serial)
Detection AP					
BUNG POINT	75.26 ± 0.58	75.76 ± 0.43	75.76 ± 0.43	74.29 ± 0.30	−0.50
GRASP POINT	61.09 ± 0.11	61.01 ± 0.52	61.01 ± 0.52	61.38 ± 0.32	+0.08
BLADDER POINT	72.43 ± 0.29	70.42 ± 0.43	70.42 ± 0.43	70.04 ± 0.95	+2.01
CUT POINT	65.72 ± 0.56	65.91 ± 0.51	65.91 ± 0.51	67.38 ± 0.30	−0.19
Keypoint AP					
BUNG POINT	78.99 ± 0.71	79.91 ± 0.68	79.79 ± 0.73	78.94 ± 0.50	−0.92
GRASP POINT	63.81 ± 0.17	64.05 ± 0.30	64.06 ± 0.32	66.35 ± 0.83	−0.24
BLADDER POINT	79.17 ± 0.64	77.64 ± 0.70	77.37 ± 0.36	76.90 ± 0.42	+1.53
CUT POINT	71.12 ± 0.37	71.07 ± 0.20	70.99 ± 0.14	73.20 ± 0.40	+0.05

## Appendix C. Data Acquisition

The National Institute of Animal Science (NIAS) provided access to its slaughter line, and all experimental data were acquired at this facility. The data for training and validation were collected on 23 and 30 June 2026, and the additional data for testing were collected on 7 October 2026. Table A3 lists the carcasses.

**Table A3:** Carcasses used for data acquisition. NIAS, National Institute of Animal Science.

Carcass	Slaughter Date and Time	Site	Partition	Frames Used	Representative Image
1	2026-06-23 13:37	NIAS	Training/validation	345	
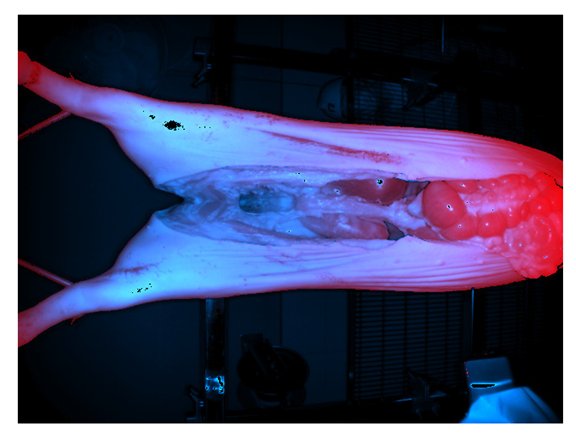
2	2026-06-23 14:18	NIAS	Training/validation	352	
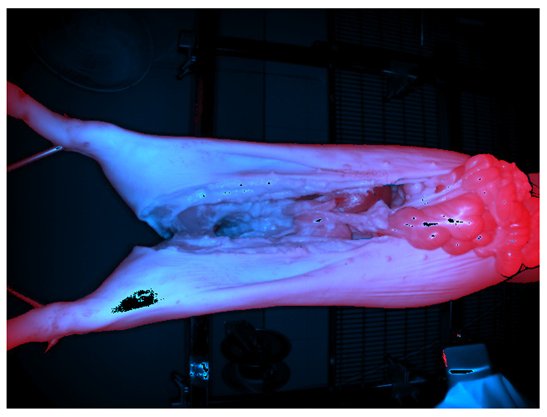
3	2026-06-23 14:57	NIAS	Training/validation	365	
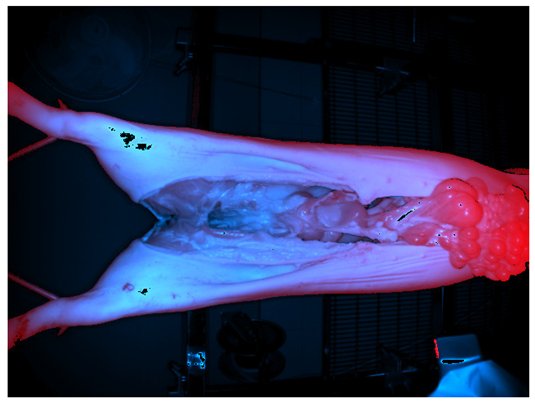
4	2026-06-23 15:39	NIAS	Training/validation	395	
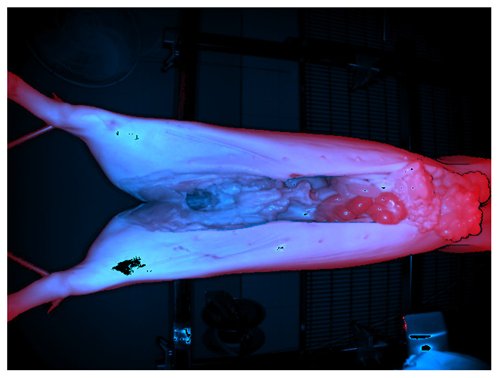
5	2026-06-30 09:41	NIAS	Training/validation	324	
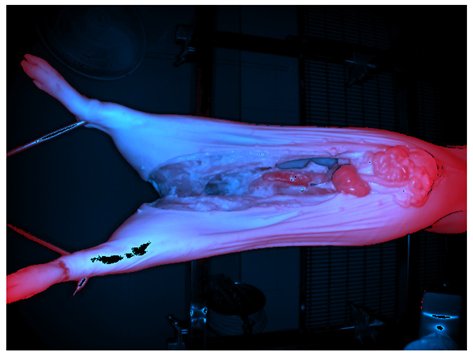
6	2026-06-30 10:10	NIAS	Training/validation	386	
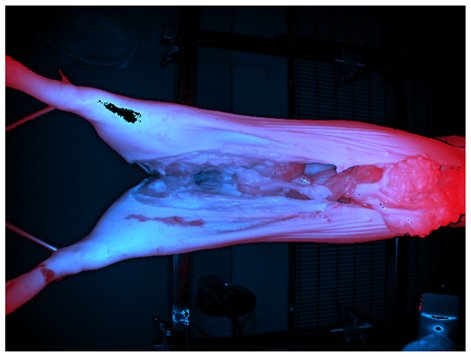
7	2026-06-30 11:00	NIAS	Training/validation	364	
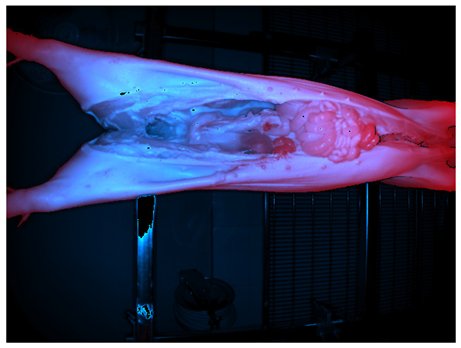
8	2026-06-30 13:37	NIAS	Training/validation	349	
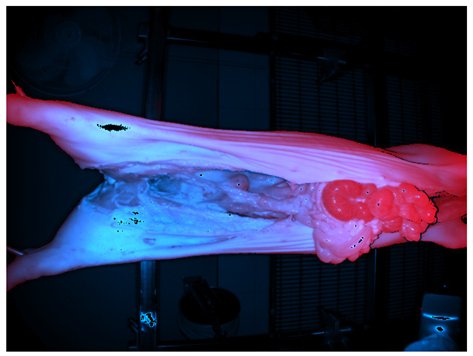
9	2026-06-30 14:44	NIAS	Training/validation	370	
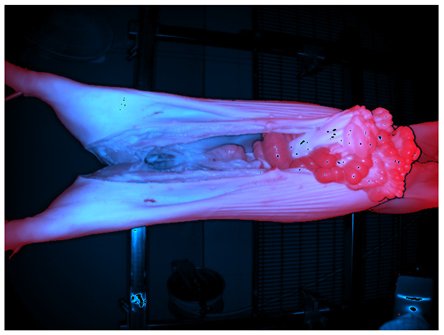
p1	2026-10-07 09:38	NIAS	Test	100	
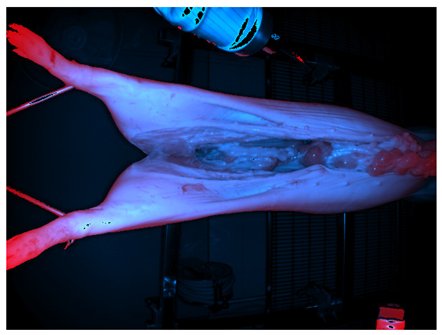
p2	2026-10-07 10:42	NIAS	Test	100	
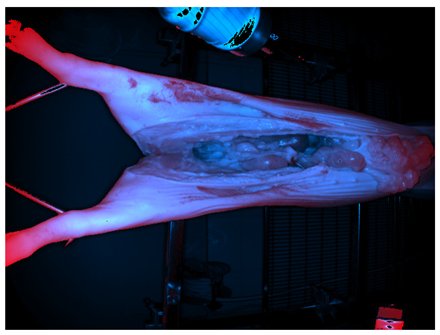
p3	2026-10-07 11:19	NIAS	Test	100	
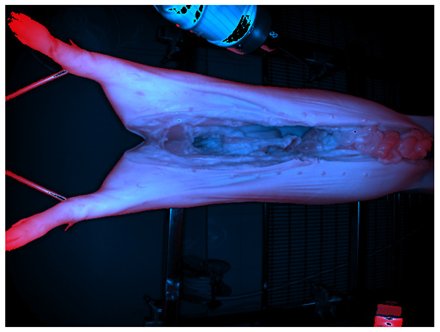
p4	2026-10-07 14:03	NIAS	Test	100	
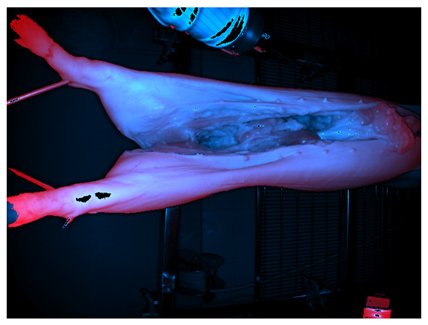
p5	2026-10-07 14:55	NIAS	Test	100	
